# Accurate Protein Dynamic Conformational Ensembles: Combining AlphaFold, MD, and Amide ^15^N(^1^H) NMR Relaxation

**DOI:** 10.3390/ijms26188917

**Published:** 2025-09-12

**Authors:** Dmitry Lesovoy, Konstantin Roshchin, Benedetta Maria Sala, Tatyana Sandalova, Adnane Achour, Tatiana Agback, Peter Agback, Vladislav Orekhov

**Affiliations:** 1 Shemyakin-Ovchinnikov Institute of Bioorganic Chemistry RAS, 117997 Moscow, Russia; 2 Science for Life Laboratory, Department of Medicine, Karolinska Institute, SE-171 65 Solna, Sweden; sala.benedettamaria@gmail.com (B.M.S.); adnane.achour@ki.se (A.A.); 3 Division of Infectious Diseases, Karolinska University Hospital, SE-171 76 Stockholm, Sweden; 4 Department of Chemistry and Molecular Biology, University of Gothenburg, P.O. Box 465, SE-405 30 Gothenburg, Sweden; tatiana.agback@slu.se; 5 Department of Molecular Sciences, Swedish University of Agricultural Sciences, P.O. Box 7015, SE-750 07 Uppsala, Sweden; 6 Swedish NMR Centre, University of Gothenburg, P.O. Box 465, SE-405 30 Gothenburg, Sweden

**Keywords:** 4D dynamical conformation ensembles, *Streptococcus pneumoniae* protein, back-calculated NMR parameters, ^15^N cross-correlated relaxation, pulse program optimization

## Abstract

Conformational heterogeneity is essential for protein function, yet validating theoretical molecular dynamics (MD) ensembles remains a significant challenge. In this study, we present an approach that integrates free MD simulations, starting from an AlphaFold-generated structure, with refined experimental NMR-relaxation data to identify biologically relevant holistic time-resolved 4D conformational ensembles. Specifically, we select trajectory segments (RMSD plateaus) consistent with experimental observables. For the extracellular region of *Streptococcus pneumoniae* PsrSp, we found that only specific segments of the long MD trajectory aligned well with experimental data. The resulting ensembles revealed two regions with increased flexibility, both of which play important functional roles.

## 1. Introduction

Over the past decade, conformational ensembles have gained increasing recognition as the most accurate representation of a protein’s native state, offering valuable insights into the fundamental relationships between protein structure, dynamics, and function [1,2]. This shift came from the realization that traditional paradigms fail to fully capture the complexity of biological functions, as they neglect the dynamic nature of proteins [3,4]. Recent advances in physics and chemistry, particularly the development of energy landscape theory, have significantly reshaped molecular biology by highlighting that proteins continuously fluctuate between multiple conformational states, each corresponding to distinct energy levels [4]. The conformational distribution is determined by energy profiles that govern the function of the molecular system under study. Obtaining a reliable 4D model (defined as a three-dimensional spatial structure evolving over time) of the most energetically favourable, and therefore most populated, region of conformational space offers a more realistic and comprehensive understanding of protein function in living systems.

For decades, the relationship between sequence, structure, and function in molecular biology was based on the assumption that each protein sequence folds into a single, averaged 3D structure under given conditions. This foundational belief deeply influenced traditional structural biology approaches and is reflected in widely used software packages for nuclear magnetic resonance (NMR) spectroscopy, such as **CNS** [5]**, XPLOR [6], CYANA [7,8], HADDOCK [9], and CS-RosettaCM [10],** originally designed to produce a single structure that satisfies all conformational averaged experimental constraints. Despite its limitations, the single-structure paradigm facilitated the creation of extensive public databases of experimentally determined protein structures, primarily obtained through X-ray crystallography, cryo-electron microscopy (cryo-EM), and NMR spectroscopy [8]. Capitalising on this data, ground breaking developments in Artificial Intelligence, such as **AlphaFold (AF),** have significantly improved the predictions of static protein structures. As structural biology shifts from studying well-defined macromolecules toward larger, more complex, and flexible molecular systems, there is an increasing need for structural approaches capable of capturing the holistic time-resolved 4D spectrum of conformational heterogeneity. This transition from static, single-structure models to dynamic ensemble representations requires the development of novel, conceptually distinct computational methods and experimental tools [11,12,13,14,15,16,17,18,19].

Solution-state NMR spectroscopy is a powerful tool for studying conformational ensembles as it inherently captures the physical properties of biomolecules averaged across multiple conformations, offering insights into protein dynamics across a wide range of timescales. From the beginning, NMR datasets, such as chemical shifts (CS) [20,21,22], residual dipolar couplings (RDCs) [12,15,23,24,25,26,27,28,29], and paramagnetic relaxation enhancements (PREs) [30,31], have been the primary choice for defining conformational ensembles.

Despite NMR’s exceptional ability to probe backbone and side-chain dynamics, relaxation measurements have been relatively underutilized in the determination of structural ensembles. Relaxation measurements, such as longitudinal (R_1_), transverse (R_2_), and heteronuclear NOE, provide detailed insights into dynamic structural ensembles, reflecting their heterogeneity and temporal properties [12,22,32,33,34,35]. Early studies employed the model-free (MF) approach [36,37,38] to estimate the rates and amplitudes of internal motions on the pico- to nanosecond timescale. The analysis yields the generalized order parameter (S^2^), which quantifies the structural range of fast internal motions (from 0, indicating complete disorder, to 1, indicating complete rigidity), and the correlation time (τ_e_), which reflects the timescale of structural fluctuations.

Interpretation of NMR relaxation data in the context of conformational ensembles remains challenging due to the difficulty in distinguishing structural features from dynamic behaviour [39,40]. However, recent advances in molecular dynamics (MD) simulations—driven by improved force fields [41,42,43,44,45] and more affordable access to high-performance computing—have enabled the integration of relaxation data with computational models. This integration allows for more accurate modelling and validation of dynamic conformational ensembles sampled on the picosecond-to-nanosecond timescale. Several strategies have been developed to combine NMR relaxation data with MD simulations to capture dynamic conformational states in solution. The original approach employs constrained MD simulations with additional force-field terms to obtain MD trajectories aligned with the experimental model-free order parameters and other NMR data [12,24,46].

Another method extracts backbone ^1^H–^15^N vector motions from an unconstrained MD trajectory calculated with the most realistic force fields, followed by back-calculation of order parameters or NMR relaxation rates [26,47,48,49,50,51,52]. Various MD force fields were benchmarked using model proteins like ubiquitin against the experimental R_1_, R_2_, and NOE-derived order parameters (S^2^) [26,53,54].

In addition to direct comparison between back-calculated and experimental relaxation parameters, several integrative methods have been developed to refine MD ensembles against NMR data. ABSURDer employs χ^2^ minimization with an entropy restraint to reweight trajectory blocks, thereby improving agreement with relaxation observables while avoiding overfitting [55]. Similarly, Bayesian and maximum entropy (MaxEnt) approaches adjust ensemble weights in a statistically rigorous fashion, ensuring minimal perturbation of the underlying MD distribution while enforcing consistency with experiments (see for reviews [46,56,57]). These approaches are powerful when the simulated ensemble is ergodic (MD trajectory is representative of the true conformational distribution), but they may obscure distinct metastable conformations by averaging across basins.

Along this line, Palmer and colleagues [21] were probably the first to demonstrate that NMR relaxation data can serve to select MD trajectories inconsistent with experimental dynamics. They showed that back-calculated NMR chemical shifts and spin-relaxation data provide complementary insights into the structure and dynamics of intrinsically disordered proteins (IDPs). Their work revealed a strong agreement between experimental and computed generalized order parameters, allowing the identification of MD trajectories that most accurately reflect experimental observations. This approach for exploring conformational ensembles in IDP [50] and global proteins [52,58] has since been applied.

The analysis was further improved by replacing experimental R_2_, which may be biased by the slow conformational exchange, with the cross-correlated relaxation (η_xy_) rates [50].

Building on this progress, we previously conducted a study validating dynamic ensembles of the Dengue II protease protein derived from unconstrained MD simulations [52]. In that work, we selected MD trajectories by comparing experimental and back-calculated relaxation parameters, including backbone R_1_, NOE, R_2_, and various types of cross-correlated relaxation in methyl side-chain dynamics. Additionally, we examined how starting molecular models, obtained from experimental methods such as X-ray crystallography and NMR-refined structures of the Dengue II protease, can serve for further refinement, structural analysis, and differentiation between conformational states.

Significant challenges remain in validating theoretical structural–dynamic ensembles, primarily due to incomplete sampling of the conformational space [46,54]. Recent studies have shown that AlphaFold holds great promise not only in predicting the “best” single structure but also in generating conformational ensembles consistent with experimental and evolutionary data [59,60,61,62,63]. AlphaFold-generated structural ensembles are considered promising starting points for MD simulations [64,65,66], as they may effectively explore a broad range of local and global energy minima.

Importantly, MD simulations are not the only route to generate computational ensembles of protein conformations. Recent developments in AlphaFold have shown that even local installations can be used to generate arbitrarily many models, which often resemble an NMR-type ensemble—structures that are highly similar yet not identical, thus reflecting conformational heterogeneity. Beyond single-protein runs, database-driven approaches have also been proposed. For instance, Lewis et al. [67] demonstrated that ensembles can be constructed by analysing AlphaFold Database entries of homologous proteins with similar sequences, thereby providing alternative conformational landscapes not sampled in a single MD trajectory. Correlating NMR relaxation data not only with MD-derived ensembles but also with AlphaFold-generated structural ensembles and database-derived models represents a promising future direction, offering a broader basis for testing the robustness of experimental–computational integration.

Parallel advances in integrative structural modelling are expanding the scope of conformational ensemble generation. Methods such as EMBuild [68], DiffModeler [69], and DEMO-EM/DEMO-EM2 [70,71] combine cryo-EM density maps with AlphaFold predictions and machine-learning-based fitting to model multi-domain assemblies with high accuracy, even at intermediate resolutions. These approaches illustrate how deep learning and experimental density data can be combined to reconstruct static or semi-flexible assemblies.

Beyond static structures, recent methods such as FoldPAthreader [72] extend AphaFold-based predictions toward folding pathways, inferring plausible intermediates using evolutionary information and fragment assembly. Together, these pipelines underscore the rapidly expanding toolkit for bridging experimental data, AI-based prediction, and dynamic ensemble modelling.

Building on these advancements, we present an efficient AlphaFold-MD-NMR based method that uses back-calculated R_1_, NOE, and η_xy_ relaxation parameters of 4D dynamical conformation ensembles of folded proteins best aligned with the experimental relaxation data.

Unlike the traditional inverse modelling approach used in protein NMR spectroscopy, which separately estimates an average structure and angular fluctuations of NH vectors while neglecting potentially dominant translational displacements, our method is based on discrete selection of theoretical 4D models: segments of MD trajectories with stable RMSD. This 4D structural–dynamic model captures a complete dynamic picture of backbone and side chains.

We also introduce an improved experimental scheme for measuring η_xy_ relaxation in backbone HN groups.

We applied this approach to the extracellular region of *Streptococcus pneumoniae* protein Psr_Sp_ (residues 131–424). Psr_Sp_ catalyses the attachment of cell wall teichoic acid and/or other polysaccharides to the peptidoglycan layer of Gram-positive bacterial cell walls, a process critical for bacterial survival [73]. P_srSp_ represents a critical antimicrobial target in Gram-positive bacteria due to its central role in maintaining cell wall architecture and overall bacterial fitness. Experimental deletion of the *psrSp* gene in *Streptococcus pneumoniae* has been shown to cause a pronounced reduction in capsule volume, a significant decline in bacterial viability over time, and severe impairment of cell wall integrity [74]. The capsule is a major virulence determinant in *S. pneumoniae*, and its disruption directly compromises the bacterium’s ability to evade host immune defences. Similarly, compromised cell wall stability renders the bacterium more susceptible to osmotic stress and host-derived antimicrobial factors. Mechanistically, P_srSp_ likely uses conformational flexible substrate-binding loops and a conformational selection mechanism to recognize and process diverse polysaccharide substrates. Structural characterization of these dynamic regions, combined with functional assays, could reveal insights into enzyme specificity, regulation, and strategies for inhibition.

Recent combined crystallographic and NMR studies of Psr_Sp_ and related LCP homologs highlighted the importance of loops surrounding the active site for substrate binding [75]. Variability in loop length and composition across species contributes to substrate specificity, with dynamic conformational changes likely playing a key functional role. Since these regions represent promising targets for antibiotic development, a deeper understanding of their flexibility and dynamics, particularly in hotspot regions, is essential for guiding future drug discovery efforts. Here, we constructed and validated a structural–dynamic model of Psr_Sp_. Our model reveals the functional mobility of two key hotspot regions: a loop at the active site and a substrate-binding pocket composed of an α -helix and an adjacent irregular segment. The resulting dynamic conformational ensemble depicts how these dynamic regions influence ligand interactions.

We present a novel AlphaFold-MD-NMR approach in NMR structural biology, enabling the generation of 4D experimentally validated dynamic conformational ensembles from accessible NMR relaxation data, without requiring costly experimental methods like X-ray crystallography or traditional NMR. By selecting MD-derived ensembles consistent with backbone (R1, NOE, η_xy_) and side-chain relaxation parameters [52], our method links high-resolution protein dynamics directly to NMR observables, complementing cryo-EM and AlphaFold-based models. While currently applied to folded proteins such as the extracellular region of Streptococcus pneumoniae P_srSp_, this approach can be extended to intrinsically disordered proteins, multi-domain assemblies, and enzyme–substrate complexes, offering a versatile tool for probing structure–function relationships, guiding drug discovery, and refining computational models of protein dynamics.

## 2. Results

### 2.1. Selection of Relaxation Parameters for MD Trajectory Verification: Comparison of R_2_ and η_xy_ Relaxation Data

**Figure 1:**
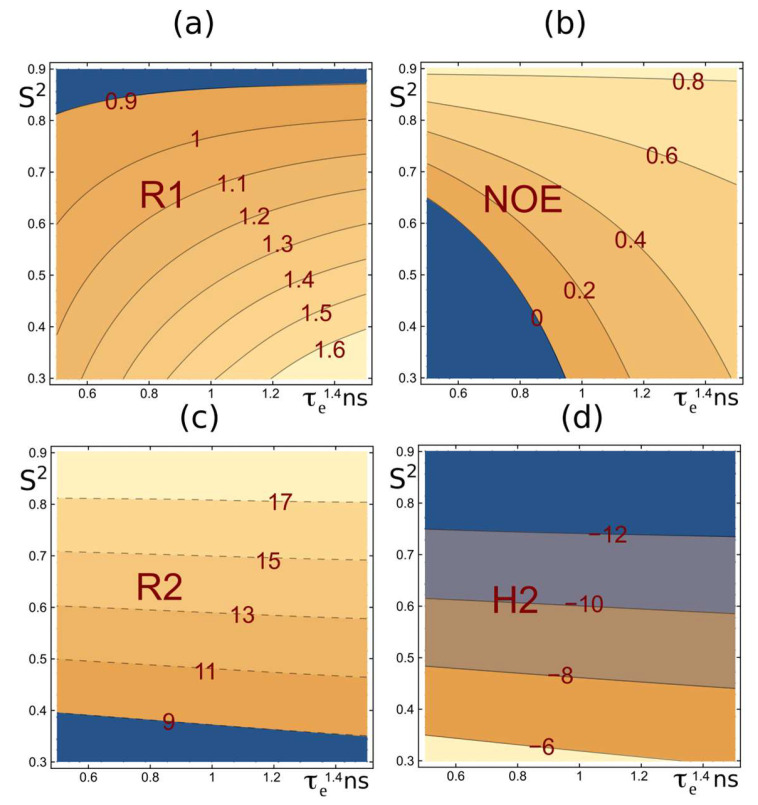
**Schematic profiles of relaxation parameters**.

Typically, the set of relaxation parameters used to characterize protein backbone dynamics includes R_1_ (Figure 1a), NOE (Figure 1b), and R_2_ (Figure 1c) [26]. However, the practical use of R_2_ relaxation rates is hindered by several systematic errors. The most significant issues are (i) the contribution of chemical exchange (R_ex_) [76,77,78], (ii) relaxation delay-dependent modulation of signal intensities by water saturation, caused by the direct exchange of amide protons and cross-relaxation-mediated coupling between protein and water magnetization, and (iii) off-resonance effects in CPMG blocks [79].

As an alternative to R_2_, one can use the η_xy_ experiment [50]. In this study, we use carefully designed measurements of η_xy_, which are free from the aforementioned issues, to verify the amplitudes of NH vectors’ angular intramolecular motions.

*R*_1_, *NOE*, *R*_2_, and *η_xy_* from Equations (1) and (4)–(7) are shown in panels (a), (b), (c), and (d), respectively, as functions of the internal motion parameters *τ_e_* and *S*^2^ = *S*^2^_fast_ × *S*^2^_slow_ (with *S*^2^_fast_ fixed at 0.9) for a protein with an overall correlation time *τ_c_* of 14.7 ns. These profiles were generated using the classical extended Lipari–Szabo model with

(1) $$J\left(\omega \right)=\frac{S_{fast}^{2}S_{slow}^{2}\tau_{c}}{1+{\left({\omega \tau }_{c}\right)}^{2}}+\frac{S_{fast}^{2}\left(1-S_{slow}^{2}\right)\tau_{e}‘}{1+{\left(\omega \tau_{e}‘\right)}^{2}}$$

and $\tau_{e}{‘=\tau }_{c}\tau_{e}/\tau_{c}{+\tau }_{e}$, which utilizes three internal motion parameters (*τ_e_*, *S*^2^*_fast_*, S^2^*_slow_*) and single overall correlation time *τ_c_* [37,80,81,82]. The plots illustrate the most typical ranges for *S*^2^ and *τ_e_*. The dashed profiles in the *R*_2_ panel (c) depict the influence of systematic experimental errors, such as *R*_ex_, on the measurements. *η_xy_*, shown in panel (d), displays a *τ_e_* versus *S*^2^ profile similar to that of *R*_2_ (panel (c)) but is free from the aforementioned issues.

**Figure 2:**
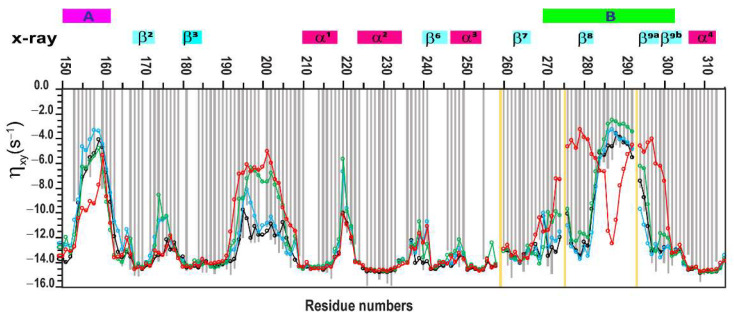
**Psr_Sp_ amide backbone ^15^N(H) η_xy_ dynamic parameters obtained on a 600MHz spectrometer**. This panel presents an extended plot of amino acid residues that ranged between 150 and 315, experimentally measured ^1^H-^15^N CSA/DD cross-correlation relaxation (η_xy_) data, as light grey solid bars. Theoretical η_xy_ values, recalculated from MD trajectory data, are shown as solid lines in black, blue, green, and red. These correspond to MD trajectory ensembles for the time segments (I) 700–1200 ns, (II) 1750–2250 ns, (III) 2500–3000 ns, and (IV) 4650–5150 ns, respectively. Theoretical and experimental errors are available in Figure S1. Theoretical errors were estimated using bootstrap analysis, as described in the Section 4. The secondary structural elements of PsrSp are shown at the top of the panels, based on the previously determined crystal structure of PsrSp proteins [75]. Yellow bars show the position of prolines. A and B loop regions of Psr_Sp_ are shown in pink and green, respectively.

The final experimentally obtained η_xy_ relaxation data, along with their fitted errors for the Psr_Sp_ protein, are presented in Table S1 and used for the validation of the MD-derived conformation trajectory segments in Figures S1–S3 and Figure 2.

### 2.2. Determination of the Isotropic Rotational Tumbling Time of Protein

The classical approach [33] for estimating rotational tumbling time (*τ_c_*) utilizes *R*_2_ and *R*_1_ values. However, as mentioned above, *R*_2_ values are affected by residual *R_ex_* contribution, CPMG off-resonance effects, and several other artefacts [76,77,78]. To address these issues in estimating *τ_c_* values, we used *η_xy_* values (Equation (4)) instead of *R*_2_. The Lipari-Szabo model [37] was applied to stable backbone NH vectors with minimal *R*_1_ and *η_xy_* experimental errors, providing two key values, *S*^2^ and *τ_c_* for all NH groups, by numerically solving Equations (4) and (5) with

(2) $$J\left(\omega \right)=\frac{S^{2}\tau_{c}}{1+{\left({\omega \tau }_{c}\right)}^{2}}$$

Using this approach, the *τ_c_* value for the Psr_Sp_ protein was estimated by selecting stable amino acids with *S*^2^ > 0.8, resulting in 76 stable NH groups, labelled in Table S1. The mean *τ_c_* value was found to be 14.7 ± 0.2 ns, supporting the predominantly monomeric form of Psr_Sp_ in solution. This experimental *τ_c_* value is consistent with predictions made using an empirical equation [33] that accounts for temperature and molecular weight. At 35 °C, the predicted *τ_c_* values were 15.6, 31.1, and 46.6 ns for the monomeric, dimeric, and trimeric forms of Psr_Sp_, respectively.

### 2.3. Identification and Validation of Structural Ensembles of the Psr_Sp_ Protein Based on Backbone Relaxation Dynamic

**Figure 3:**
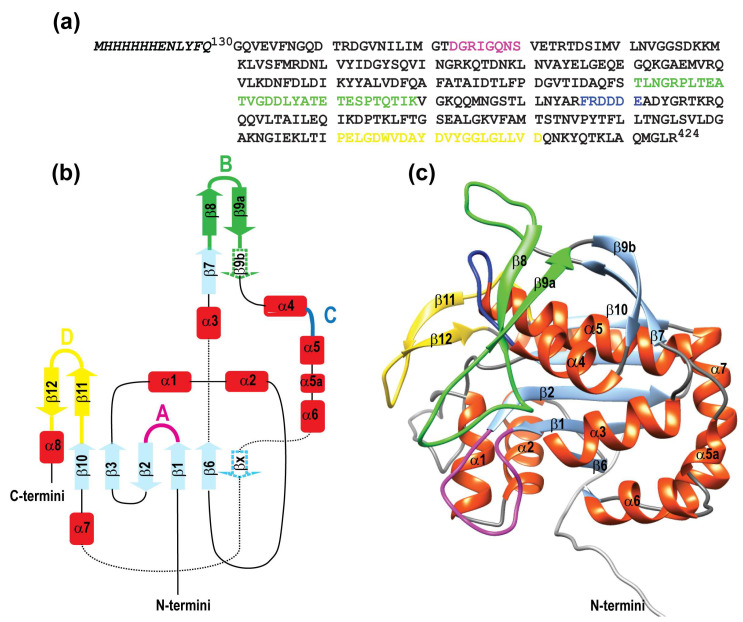
**Topology diagram of Psr_Sp_ protein**. (**a**) Amino acid sequence of the Psr_Sp_ construct; the color cording corresponds to A–D reggions in (**b**) respectively. The TAG aa is indicated in italic. The topology diagram of Psr_Sp_ is shown on the (**b**) left panel while a cartoon representation of the AlphaFold3 molecular model of the extracellular region of Psr_Sp_ is shown on the right (**c**). Panel coloured according to the topology diagram. As for colouring, all α -helices are red; β-strands are light blue, except β11 and β12, which are yellow, and β8 and β9 a,b, which are green; linkers are black, except for loops and linkers belonging to the four regions A–D that are suggested to be important for substrate binding to Psr_Sp_ are coloured in pink, green, dark blue, and yellow, respectively.

We have recently determined the crystal structure of the extracellular region of the *Streptococcus pneumoniae*-associated polyisoprenyl-teichoic acid-peptidoglycan teichoic acid transferase Psr_Sp_ (residues 131–424) [75], whose topology diagram is presented in Figure 3b. Based on the near-complete assignment of the ^1^H, ^13^C, and ^15^N backbone resonances, as well as the ^13^Cβ side chain resonances for the Psr_Sp_ domain (for the amino acid sequence of the Psr_Sp_ construct, Figure 3a), the secondary structure of Psr_Sp_ in solution was determined and compared with the high-resolution X-ray crystal structure. Additionally, dynamical S^2^ predicted order parameters were extracted and compared with structural information and the crystallographic B-factor, allowing us to qualitatively evaluate the flexibility of this protein. Although the assignment of the backbone resonances is an essential first step, this experimental data alone are insufficient to extract the conformational ensembles reflecting the flexibility of the protein.

To comprehensively explore the conformational space and generate an ensemble of Psr_Sp_ conformations, we employed a strategy previously developed by our team [52]. This approach leverages molecular dynamics (MD) trajectories validated against experimentally determined relaxation parameters of the NH backbone. Notably, in this protocol, the η_xy_ relaxation parameter was used in place of R_2_ to enhance accuracy. In comparison to our previously established protocols [52], which utilized NOE-based NMR and X-ray structures as starting points, the present study explores the validation of a novel approach where an AlphaFold-generated structure serves as the starting structure for free MD simulations. The motivation for testing this method lies in the accessibility of AlphaFold, which can generate both single molecular models and conformational ensembles [61,83,84,85], offering a faster and more cost-effective alternative to traditional NMR or X-ray structures for initiating MD simulations. This approach enables the generation of trajectory intervals used to back-calculate the relaxation parameters R_1_, η_xy_, and NOE for the Psr_Sp_ ^1^H-^15^N amide backbone. The AF-predicted three-dimensional structure of the Psr_Sp_ protein, used as the starting point for a 6 μs free MD simulation, is presented in Figure 3c. In particular, the root mean square deviation (RMSD) between the AF structure used in this study and the experimentally determined X-ray structure of Psr_Sp_ [75] is 1.32 Å for 277 aligned residues. The N-terminal 14 residues, loop A, and approximately 10 residues following helix 5 Figure 3c are excluded from alignment due to significant positional deviations.

The selection of MD trajectory intervals and their length for back-calculating relaxation parameters (R_1_, η_xy_, and NOE) is ambiguous and remains a subject of discussion. Typically, stable trajectory segments, defined by low RMSD values, are used for such analyses. In this study, we examine the validity of the RMSD-based criterion. After 700 ns of free MD equilibration, the RMSD of backbone heavy atoms for dynamically stable residues of PsrSp exhibited two sharp transitions (exceeding 1.5 Å and 3.5 Å, respectively), followed by plateau regions where RMSD fluctuated around 1 Å (Supplementary Figure S4). The choice of specific time intervals within these plateaus is somewhat arbitrary. Here, we applied a single criterion: each selected interval must be 500 ns in length. The rationale for this choice is described in Methods Section 4.6.

Four distinct 500 ns time intervals were selected for the back-calculation of the relaxation parameters R_1_, η_xy_, and NOE of the PsrSp protein. These intervals define ensembles (I), (II), (III), and (IV), corresponding to trajectory segments 700–1200 ns, 1750–2250 ns, 2500–3000 ns, and 4650–5150 ns, respectively.

The first interval (I), with RMSD consistently below 0.5 Å, is located on the plateau preceding the first sharp transition at approximately 1400 ns. The second (II) and third (III) intervals, each with RMSD values below ~1.0 Å, lie within the second plateau. The fourth interval (IV) was chosen after a sharp transition at ~4100 ns. Although the region between 4100 and 6000 ns is generally unstable, it was possible to extract one 500 ns segment (4650–5150 ns) with RMSD around 1 Å that was chosen for analysis.

The back-calculated theoretical versus experimental ^1^H-^15^N R_1_, η_xy_, and NOE parameters for the Psr_Sp_ backbone are shown for all four trajectories in Figures S1–S3. The secondary structure elements based on the crystal structure of Psr_Sp_ [75] are presented at the top of Figures S1–S3.

The dynamic parameters ^15^N R_1_, η_xy_, and NOE show good agreement across all three back-calculated trajectories and with experimental data for the most stable secondary Psr_Sp_ structure elements determined by X-ray crystallography [75]. As shown in Figures S1–S3, this is evident for the β1–β7 beta strand and the α1, α2, α4, and α8 helices, where the differences between the calculated curves and experimental data fall within the error margin (Figure S1 and Figure 3b–e). This consistency indicates absence of the systematic shifts between theoretical and experimental results, validating the back-calculation protocol presented in this study.

Due to missing or highly uncertain experimental data, validation of back-calculated trajectory segments I–IV was not possible for the α3, α5, and α7 helices. As previously reported [75], resonances from these amide protons were not detected because of exceptionally slow deuterium-to-proton exchange. However, these regions in all four trajectories closely match the X-ray structure of PsrSp, so the absence of experimental data does not affect the selection of the most representative conformational ensemble.

There is good agreement between the calculated ^15^N R_1_, η_xy_, and NOE values and the experimental data in two regions of interest, A and B (Figures S1–S3), indicating high amide mobility in the loop regions. This agreement holds despite differences in the contribution of individual relaxation parameters across the trajectories. Notably, this is supported by the observed drop in NOE values from approximately 0.8 to 0.2, and a decrease in η_xy_ rates from 14 to around 3 s^−1^.

The main differences in the ^15^N R_1_, η_xy_, and NOE parameters calculated for trajectory segments (I)–(IV) are observed in the disordered segment spanning residues 190–210, as well as in the residue ranges 135–145 and 265–275, which correspond to loop regions A and B, respectively (Figures S1–S3). These discrepancies between the trajectories and the experimental data are clearly illustrated in Figure 2, which presents an extended plot of amino acid residues from 150 to 315. As previously noted, the experimental η_xy_ relaxation data do not include contributions from slow exchange R_ex_, which is expected in the loops of regions A and B. A detailed examination of these regions shows that trajectory segments (I) and (II) align more closely with the experimental η_xy_ data, with differences between experimental and calculated values remaining within one standard deviation, as shown in Figure S1b,c.

Trajectory segment (IV) shows the poorest fit to the experimental data across loop regions A, B, and D, as well as in the disordered segment spanning residues 190–210. In these regions, the differences between experimental and calculated values reach up to three standard deviations (Figure S1e). Notably, the outlier residues occur in continuous stretches rather than as isolated points.

The most intriguing result was observed for trajectory segments (II) and (III), both of which belong to the same RMSD plateau (Figure S4). While segment (II) provides the second-best fit to the experimental data after segment (I), segment (III) shows a poor fit in the disordered segment spanning residues 190–210 (Figure S1d).

To evaluate differences between experimental and calculated relaxation parameters obtained from trajectory segments (I)–(IV), the Mann–Whitney U test [86] was applied to the η_xy_ data sets. The resulting *p*-values obtained on η_xy_ data sets were as follows: 0.058155 for segment (I), 0.02915 for segment (II), and 0.000149 for segment (III). For segment (I), *p* > 0.05, indicating no significant difference between the experimental and calculated η_xy_ data. In contrast, for segments (II) and (III), *p* ≤ 0.05, indicating statistically significant differences between the experimental and calculated values. However, the *p*-value for segment (II) is still close to that of segment (I), which is consistent with the observation that most Δ-values fall within one standard deviation (Figure S1b,c). A direct comparison between trajectory segments (II) and (III) yields a *p*-value of 0.029219, further confirming a significant difference between these two data sets.

**Figure 4:**
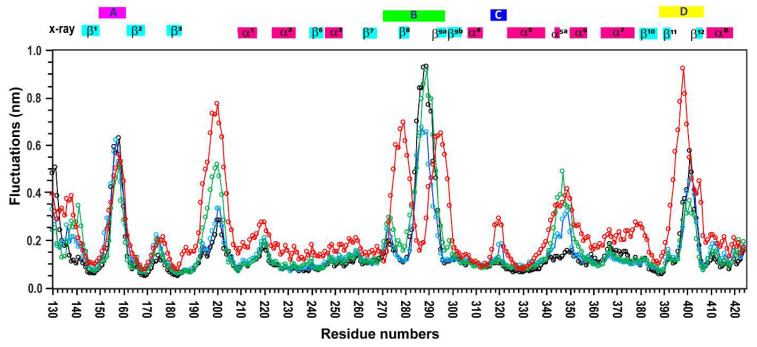
**RMSF of PsrSp calculated from MD trajectories**. The RMSF values, averaged over Cα atoms of PsrSp, are shown as line plots in black, blue, green and red for trajectory segments I (700–1200 ns), II (1750–2250 ns), III (2500–3000 ns), and IV (4650–5150 ns), respectively. Residue numbers are displayed on the x-axis, and Cα fluctuations (in nm) are shown on the y-axis.

Next, RMSF (root mean square fluctuation) analyses were performed on the MD trajectories to identify regions of structural stability and flexibility. The averaged RMSF profiles for trajectories (I)–(IV) are shown in Figure 4.

The β-sheet core, including strands β1, β2, β3, β6, β7, and β10, as well as α-helices α1, α2, α3, and α4, remained highly stable across all simulations, with fluctuations below 2 Å. These findings, along with relaxation data (Figures S1–S3), suggest low mobility and strong structural integrity in these core regions.

In contrast, larger fluctuations were observed in the connecting loops of regions A, B, C, and D, and in the disordered segment spanning residues 190–210. Notably, the A loop showed fluctuations up to 6 Å, and similar dynamic profiles were observed across all four conformational ensembles. However, important differences emerged in the flexible regions.

Specifically, ensembles (I), (II), and (III) generally followed a similar fluctuation pattern throughout the protein sequence, except in the α5a helix, where ensembles (II) and (III) exhibited greater variability (up to 4 Å) compared to ensemble (I).

Ensemble (IV) showed the highest fluctuations in the 190–210 region and in loop D. Remarkably, in region B, ensemble (IV) split into two subpopulations with strong fluctuations (~8 Å), whereas ensembles (I)–(III) exhibited a single, more uniform set of fluctuations.

These deviations in ensemble (IV) are further supported by predicted relaxation properties indicating increased dynamics that do not align with experimentally measured relaxation parameters (Figure 2). In contrast, ensembles (I)–(III) show fluctuation patterns that are consistent with both experimental and predicted dynamic data.

### 2.4. Validation of Structural Ensembles of the PsrSp Protein by Alternative Methods

One way to validate a conformational ensemble is through complementary structural methods. The recently determined crystal structure of PsrSp [75] revealed three protein molecules in the asymmetric unit. These three monomers (A, B, and C) exhibit highly similar overall structures, with differences mainly observed in the conformations of several active site loops that are likely involved in substrate binding.

In this study, we compared three subunits (A, B, and C) from the crystal structure of PsrSp [75] with the most populated structures from MD trajectory segments (I)–(IV), obtained via segment cluster analysis. RMSD values are presented in Table 1.

Trajectory segment (I) reveals a stable protein core comprising ~240 residues, maintaining structural integrity with an RMSD of ~1 Å. This suggests that the crystal structures of subunits A, B, and C of PsrSp fall within the conformational ensemble captured in this simulation.

**Figure 5:**
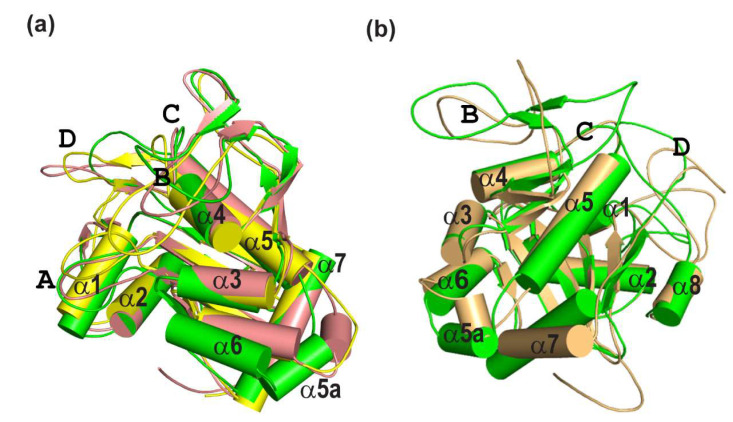
**Structural–dynamic models of the PsrSp amide backbone.** (**a**) Superposition of the most representative models of PsrSp from three MD trajectory segments, (I) 700–1200 ns (green), (II) 1750–2250 ns (pink), and (III) 2500–3000 ns (yellow), illustrates the mobility of α-helices 5a and 6. (**b**) Superposition of representative models from trajectory segments—(I) 700–1200 ns (green) and (IV) 4650–5150 ns (brown)—reveals additional mobility of α-helices 5a, 6, and 7, as well as structural disorder in the B region. In trajectory (IV) (4650–5150 ns), the β-sheet composed of strands β8 and β9a is completely disordered, in contrast to trajectory segments (I), (II), and (III).

In contrast, analysis of segments (II) and (III), limited to 201–222 aligned residues, shows increased RMSD values of 1.5–1.7 Å. Structural superposition (Figure 5a) reveals shifts in several helices, α5a and α6, compared to segment (I).

Segment (IV) also retains a stable core (~200 residues), but loop regions adopt alternative conformations. Notably, small hairpins β8 and β9a in loop B, seen in the crystal structure are absent. Furthermore, helices α5a and α6, which begin to shift in trajectory segments (II) and (III), undergo further displacement in segment (IV) (Figure 5b).

These observations suggest that the conformations observed in trajectories (II), (III), and (IV) are not fully supported by complementary validation methods such as X-ray crystallography.

Next, we attempted to cross-validate the ensembles from trajectories (I)–(IV) by comparing experimentally measured chemical shifts of the backbone atoms (Cα and C=O) with those recalculated from the conformational ensembles using ShiftX2 v1.10 chemical shift prediction software [87].

The results are promising in part: the predicted chemical shifts differ between ensembles (Figure S5), particularly in regions where the structural ensembles show the greatest discrepancies. However, comparison with experimental data remains inconclusive and does not allow clear discrimination between conformations.

These findings highlight the need for further advancement in chemical shift prediction methods to improve the accuracy of structural ensemble validation in proteins.

## 3. Discussion

One of the most ground-breaking advances in structural biology over the past decade has been the recognition of the pivotal role that conformational heterogeneity plays in key biological processes. Recent reviews have highlighted diverse systems and mechanisms that underscore the significance of dynamic conformational ensembles [4,17,88]

Protein flexibility, particularly in ligand binding [44,89,90,91,92], is vital for modulating activity through mechanisms such as shifts in conformational balance, formation of ligand-induced conformations, and active site mobility required for allosteric signalling [93]. These findings highlight the limitations of static models in capturing dynamic interactions and underscore the necessity of adopting dynamic perspectives in structural biology [3,94,95]. Efforts to generate accurate protein conformational ensembles have led to the development of advanced methodologies. Cryo-EM, while provides conformation ensembles of large protein complexes and biological machines, often suffers from resolution limitations. All-atom molecular dynamics simulations have significantly advanced the theoretical exploration of dynamic ensembles for biologically relevant systems [41,42,43,44]. Meanwhile, AlphaFold can predict structural ensembles, although the biological relevance of these ensembles requires further validation [61,83,84]. Despite these advancements, a critical challenge remains: experimentally validating these theoretical conformational ensembles.

### 3.1. Experimental Validation of Conformational Ensembles

NMR spectroscopy, particularly residual dipolar coupling (RDC) [96] and NOE-based and chemical shift (CS) methods, offers potential solutions for validation of conformation ensembles. Conformers obtained through AlphaFold, cryo-EM, or MD trajectories can be validated by back-calculating the chemical shifts of backbone and side-chain nuclei and comparing these with experimentally determined values. Software, such as ShiftX2 v.1.10 [87], provides a comprehensive chemical shift prediction package based on PDB structures, enabling the assessment of structural ensembles through these comparisons. However, the relatively low accuracy of existing chemical shift prediction methods limits the reliability of this approach for discriminating between different conformational ensembles. Protein structures obtained by traditional NOE-based NMR method generally align well with X-ray crystallography data. Careful quantitative use of ^1^H–^1^H NOEs has been employed to generate structural ensembles of small sized protein systems [97,98,99,100]. However, large, deuterated proteins and intrinsically disordered proteins (IDPs) pose unique challenges due to their flexible and heterogeneous landscapes. While NOE-based conformational ensembles provide valuable insights into protein dynamics, they come with notable limitations.

A key distinction between these methods lies in their sensitivity to conformational variability and the nature of the parameters being measured. The use of ^1^H–^1^H proton cross-relaxation (^1^H–^1^H NOE) as a criterion for constructing a network of intramolecular contacts often results in conformational bias and degeneracy because of the short-range nature of the ^1^H–^1^H NOE interactions rapidly diminishing with the proton–proton distance (R^−6^ dependency). This limitation hinders the experimental derivation of reliable ^1^H–^1^H NOE-based conformational ensemble.

Furthermore, back-calculations of ^1^H–^1^H NOE to validate all-atom MD trajectories remain challenging. Unlike ^1^H–^15^N relaxation, which is described primarily by local dipole–dipole interactions and chemical shift anisotropy (CSA), mainly reflecting fluctuations in orientation the ^1^H–^1^N vector, ^1^H–^1^H NOE is also affected by the spin-diffusion and fluctuations in inter-proton distances. Moreover, measured ^1^H–^1^H NOE values are influenced by slow conformational exchange and exchange of labile protons with water, making them poorly suited for validating dynamic ensembles.

In practical applications, NOE values represent time-averaged conformational structure, which fail to capture temporal fluctuations in protein structure. However, the aim of our study is to establish a quantitative rather than qualitative criterion for this purpose.

### 3.2. Relaxation-Based Validation of Conformational Ensembles

As an alternative, in our recent work on the NS3_pro_-NS2B protein [52], we demonstrated that the combination of MD simulations with NOE-derived restraints led to poor agreement with relaxation experimental data, resulting in inaccurate conformational ensembles. To address these limitations, we introduced a relaxation filter approach that integrates experimental relaxation measurements with unconstrained MD simulation and back-calculations of relaxation parameters.

In the present study, we show that CSA/DD cross-correlation relaxation rates, η_xy_, which, unlike R_2_, are free from contributions of in millisecond-time-scale conformational exchange and avoid other experimental artefacts, fit well with back-calculated relaxation data. We also present an improved version of the pulse sequence for measuring η_xy_, which is sensitive, free from water saturation effects on the amide proton, and optimized for large proteins. The AF-free-MD-NMR-based method proposed in this study enables robust analysis of protein dynamics and experimental validation of the conformational ensembles.

Based on the crystallographic data from our earlier studies, we highlighted the highly dynamic nature of the extracellular region of the *Streptococcus pneumoniae* protein Psr_Sp_ [75]. In this study, Psr_Sp_ (residues 131–424) was used as a model system to demonstrate the described above approach for obtaining and validating conformational ensembles. We report, for the first time, the relaxation dynamic parameters R_1_, NOE, and η_xy_ for Psr_Sp_ (Table S1 and Figures S1–S3). These experimental data provide insights into the dynamic behaviour of Psr_Sp_ in its relaxed, ligand-free state. First, we determined the overall correlation time of the protein and confirmed that Psr_Sp_ behaves as a monomer in solution. This result aligns well with our X-ray crystallography data [75]. The next step involved back-calculating the R_1_, NOE, and η_xy_ relaxation parameters for Psr_Sp_ to identify the conformational ensembles obtained from free MD simulations that best align with the experimental data. In our previous study [52], we used the X-ray structure of the Dengue II NS3-NS2B enzyme and three NOE-refined NMR structures as starting points. Here, we explored an alternative approach by using a single AlphaFold-generated structure of PsrSp as the starting point and conducting one long free MD simulation. To our knowledge, our approach, for the first time, provides experimentally verified conformational ensembles based only on measurements of the R_1_, η_xy_, and NOE relaxations without using any other additional experimental data.

Although for Psr_Sp_ we identified stretches of the MD trajectory that fit well with the relaxation data, in other cases it may happen that none of the ensembles generated in a long trajectory fit the experiment. This could be due to inappropriate starting structures, simulation conditions, or force fields, leading to incorrect sampling of the conformational space. Such MD trajectories should be discarded, and new ones produced to address these issues. For example, it is possible to start the simulation from another structure offered by AlphaFold. Nonetheless, if a conformational ensemble matches the experimental relaxation data, it may be considered as a plausible experimentally verified solution.

### 3.3. Identification of Conformational Ensembles

In this work, four distinct conformational ensembles (I–IV) with varying population distributions were obtained from a single relatively long 6 µs MD simulation. The subsequent comparison of the relaxation parameters back-calculated from these ensembles with the experimental values (Figure 2 and Figures S1–S3), revealed that ensemble I, corresponding to the trajectory segment between 700 and 1200 ns, provided the best fit to the experimental data. The differences between ensembles I and II were primarily reflected in the rearrangement of the α6 and α5a helices (Figure 5) and in the population distributions of the 10 clusters derived from the trajectories (Table S2). Notably, flexibility in these regions was suggested in the crystal structure of Psr_Sp_, where residues 342–348 adopted different conformations in three monomers present in the asymmetric unit of the crystal [75]. Furthermore, residues corresponding to helix α6 are not observed in the electron density maps of all five available crystal structures of the homologous TagT protein from *Bacillus subtilis* [101,102,103]. This points to the mobility of this region and potentially hints at its functional significance.

### 3.4. Experimental Validation and Functional Implications

Figure 4 highlights the high sensitivity of the approach proposed in this study, demonstrating that even subtle population shifts can be detected through recalculated RMSF parameters. These findings underscore the importance of experimental validation of MD trajectories.

In this study, trajectory segment (I) (700–1200 ns) showed the best agreement with experimental data. This conformational ensemble was further validated using an orthogonal approach, by comparing it with the crystal structures of PsrSp subunits.

In trajectory segment (I), regions A and B of Psr_Sp_ exhibit significantly higher flexibility than other parts of this key LCP protein. We speculate that this flexibility in loops A and B is functionally significant, potentially enabling Psr_Sp_ to catalyse the attachment of a wide range of glycopolymers to peptidoglycan.

In contrast, trajectory segments (III) (2500–3000 ns) and (IV) (4650–5150 ns) emerged as significant outliers. Both statistical comparisons of back-calculated relaxation parameters with experimental data and structural inconsistencies with crystallographic models indicated poor agreement.

RMSF values from trajectories (III) and (IV) (Figure 4) indicate flexibility in regions where neither the new relaxation experiments nor published crystallographic data support such dynamic behaviour. Based on these discrepancies, the conformational ensembles from segments (III) and (IV) should be excluded as representative states and are likely artefacts of the MD simulation.

This outcome is not unexpected, as long-timescale MD simulations, especially involving large proteins, can lead to its partial refolding, particularly given the limitations of current force fields. Such artefacts may emerge despite overall system stability.

Nevertheless, the presence of these conformations, (III) and (IV), as minor populations, potentially below the detection threshold of experimental relaxation measurements, cannot be entirely ruled out.

### 3.5. Deposition of Data and Structures

Finally, cluster analysis of the free MD simulation trajectories yielded 10 final structures for each of the two conformational ensembles, (I) and (II) (Figure S6). These structures of trajectory segment (I) have been deposited in the Protein Data Bank (PDB) (Entry ID: 9A9G) along with their S^2^ parameters, population values, and their R_1_, η_xy_, and NOE relaxation data, experimentally obtained and back-calculated from free MD simulation in the Biological Magnetic Resonance Data Bank (BMRB 52556). These data provide a valuable resource for further research, including the development of novel antibiotics targeting this essential protein.

## 4. Methods

### 4.1. Sample Preparation

All NMR experiments were performed on a U-[^15^N,^13^C,^2^H] labelled sample of Psr_Sp_. The sample preparation has been fully described in our earlier publication [75]. In short, the Psr_Sp-_ sequence was cloned into a pET28 vector in frame with a N-terminal His-tag such that 12 amino acid residues (MHHHHHHENLYF) were added; thus, the construct contains 308 residues and has a molecular mass of 35.5 kDa. The plasmids were transformed into BL21 (DE3) pLys *E. Coli* strain and cells were cultured at 37 °C in isotope ^2^H, ^15^N, and ^13^C-labelled M9 medium. Chemicals for isotope labelling (ammonium chloride, ^15^N (99%), D-glucose, ^13^C (99%) and deuterium oxide were purchased from Cambridge Isotope Laboratories, Inc(Tewksbury, MA, USA).

Protein transcription was initiated by 0.5 mM isopropyl-β-D-1-thiogalactopyranoside (IPTG) to the culture after lowering the temperature to 20 °C for overnight incubation. After centrifugation, cells were suspended in lysis buffer (20 mM Tris pH 7.5, 250 mM NaCl, 20 mM Imidazole) supplemented with complete protease inhibitor (Roche, Basel, Switzerland). Cells were lysed by sonication and cell debris was pelleted by centrifugation. The supernatant was loaded on a 5 mL HisTrap FF column (Cytiva, Marlborough, MA, USA) and the protein was eluted with 500 mM imidazole and concentrated down to 5 mL with an Amicon (Rahway, NJ, USA) Ultra centrifugal filter. The final Psrsp130–424 protein sample was exchanged into 25 mM sodium phosphate (Na_2_HPO_4_ ^+^NaH_2_PO_4_) PO_4_^3−^ giving pH 6.8, 100 mM NaCl, 1 mM NaN_3_, and 10% (*v*/*v*) D_2_O using PD10 desalting columns (GE Healthcare, Chicago, IL, USA). Protein concentration was ~0.7 mM, and spectra were acquired in a 3 mm tube. For NMR experiments, 0.1 mM DSS was added as the ^1^H chemical shift standard; ^13^C and ^15^N shifts were referenced indirectly using standard frequency ratios.

### 4.2. NMR Experiments

NMR experiments were acquired on Bruker Avance III spectrometers operating at 14.1 T, corresponding to 600 MHz, equipped with a 5 mm cryo-enhanced QCI-P probe. To improve relaxation parameters of the Psr_Sp_, the experiments were performed at 308 K temperatures. Backbone resonance assignments for Psr_Sp_ were obtained [75] and previously submitted by us to the BioMagResBank with accession code **52556**. Data were processed by TopSpin 4.06 (Bruker, Billerica, MA, USA) and analysed using CcpNmr2.4.2 [104] and Dynamics Center 2.8 (Bruker, Billerica, MA, USA).

### 4.3. Pulse Sequence for ^1^H-^15^N CSA/DD Cross-Correlation

**Figure 6:**
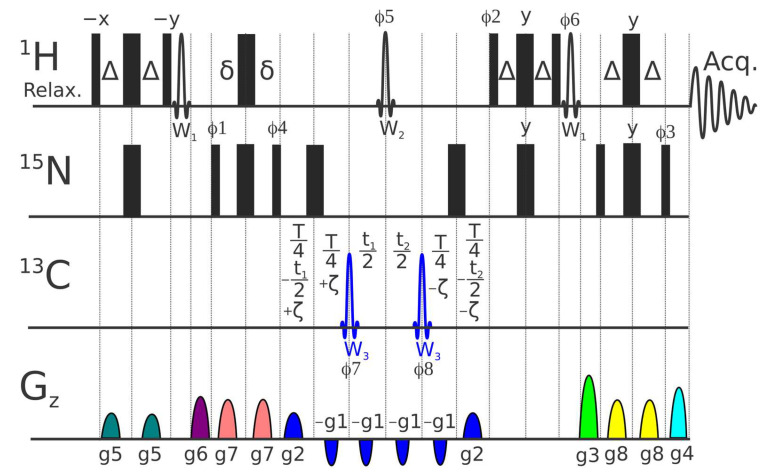
The pulse sequence of pseudo 3D ^1^H-^15^N proton-detected experiment for measurement of ^1^H-^15^N CSA/DD transverse cross-correlation relaxation in backbone ^1^H-^15^N groups. Constant-time (T period) ^15^N echo–Anti-echo (EA) sampling is implemented with the aid of t1 and t2 = t1 × (T − 4ζ)/(T + 4ζ) delays and g1×EA1, g2×EA2, g3×EA3 and g4×EA4 gradients with classical EA1 = EA2 = (1, 0.875), EA3 = (0.6667, 1), EA4 = (1, 0.6595) values; ^1^J_NH_ evolution delays were Δ = 2δ = 1/(4^1^J_CH_); constant time transverse relaxation delays, T is 60 ms; ζ delays vary from −T/4 to +T/4 modulating transverse relaxation from TROSY to antiTROSY under investigation. Narrow and thick bars represent 90° and 180° pulses, respectively; W_3_ is a 500 μs long both ^13^C^a^ and ^13^C^o^ inversion adiabatic Chirp (Crp60, 0.5, 20.1 for 600 MHz spectrometer) pulse with offset at 95 ppm; W_2_ is a 1500μs (for 600 MHz spectrometer) long 180° Reburp pulse (offset at Hn centre = 8.65 ppm); W_1_ is a 1000 μs long 90° Sinc “down” water flip-back pulse (offset at 4.7 ppm). The default phase is x and the phase cycle is: φ1 = 4 (45°, −45°); φ2 = 90°; φ3 = 90°; φ4 = 4 (0°, 180°); φ5 = 2 (0°, 0°, 180°, 180°); φ6 = −90°; φ7 = (0°, 0°, 0°, 0°, 180°, 180°, 180°, 180°); φ8 = (180°, 180°, 180°, 180°, 0°, 0°, 0°, 0°); φrec = 4 (0°, 180°). For each EA successive value φ2, φ3, and φ6 and for t1 value φ1, φ4 and the phase of the receiver are incremented by 180°, respectively. Gradient pulses with squared sine shape (SMSQ10.100) and 1 ms length except for g1 (0.5 ms) are used. The g1-g8 gradient strengths are as follows: 40%, 40%, 60%, 60.26%, 57%, 47%, 67%, and 37% whereas 100% corresponds to ca 53 G/cm. Similar to the original constant time experiment [105], the ^1^H signal position in all planes is the same and corresponds to TROSY peak, whereas ^15^N signal position ν^15^N-^1^J_NH_*ζ*2/T is a function of ζ delay (varying from −T/4 to +T/4).

A set of pulse experiments for measuring backbone ^1^H-^15^N chemical shift anisotropy/dipole–dipole (CSA/DD) cross-correlations in proteins, usually called η_xy_, have been presented previously [105,106]. The scheme, where the ^15^N chemical shift evolution and modulation of signal intensities by cross-correlation are combined during a constant time period, was shown to provide superior signal-to-noise ratio [105]. The new pulse sequence presented in Figure 6 incorporates several features designed to minimize systematic errors in this approach:

(a) We found that application of ^15^N coding echo–anti-echo gradients (g1 and g2) across all six intervals, where CSA/DD effects and sampling occur, minimizes the possible systematic errors of shaped and hard pulses with defocusing of residual undesired coherences.
(b) A classical ST2-PT TROSY block [107,108] with g1, g2, g3, and g4 echo–anti-echo gradients and φ2 and φ3 phases are used for sampling and enhanced filtering of the TROSY component and simple preliminary selection through 2δ evolution.
(c) A ^1^H Reburp inversion pulse (W_2_), selective on amide protons, preserves water magnetization along the +Z-axis and ensures uniform water (saturation) state for all ζ delays.
(d) W_2_ additionally ensures uniformity with respect to the ^2^J_N-Hα_ scalar coupling evolution across all ζ delays. Note that a non-negligible fraction of Hα protons are typically present even in deuterated protein samples.
(e) All water flip-back pulses (W_1_) are placed outside the periods of the magnetization transfer over the ^1^J_NH_ coupling and are followed by gradients.

### 4.4. Determination of ^1^H-^15^N CSA/DD Cross-Correlation (η_xy_), Relaxation Rate R_1_ and ^1^H-^15^N Nuclear Overhauser Effect (NOE)

Backbone relaxation parameters R_1_ were recorded in pseudo 3D mode with randomized and interleaved [37] relaxation delays using a standard Bruker pulse sequence, TROSY-version modified by Bax and colleagues in [109] trt1etf3gpsitc3d.3. Spectral widths (SW^1^H) of 16 ppm over 1024 complex points in the ^1^H dimension and spectral widths (SW^15^N) of 40 ppm and 128 complex points in the ^15^N dimension with 32 transients (NS) were used. R_1_ values were determined from a series of 11 relaxation delays: 10, 90, 192, 260, 380, 480, 690, 980, 1220, and 1444 ms. The errors in the R_1_ experiment were defined by an exponential decay fitting with cut-off at 5%.

Backbone ^1^H-^15^N steady-state heteronuclear NOEs were measured using TROSY-type experiments [110] implemented in Bruker pulse sequence, trnoeetf3gpsi3d.3. Two-dimensional experiments, including acquisition of NOE-enhanced and unsaturated spectra, were collected using D1 = 1 s with a follow up ^1^H saturation time of 3 s, spectral widths (SW^1^H) = 16 ppm and 1024 complex points in the ^1^H dimension, and (SW^15^N) = 40 ppm with 256 complex points, NS = 32. NOE values were obtained by dividing ^1^H-^15^N peak intensities in an NOE-enhanced spectrum by the corresponding intensities in an unsaturated spectrum, with an error defined by the software Dynamics Center 2.8 (Bruker, Billerica, MA, USA) with cut-off set at 5%.

A complete set of ^1^H-^15^N CSA/DD cross-correlation relaxation rates, η_xy_, for the backbone amides was acquired at 600 MHz utilizing the pulse program presented above in Figure 6. Experiments were performed using NS = 24 on a time domain grid of 1 K × 128 complex points with spectral width/acquisition time of 15 ppm/114 ms for ^1^H and 40 ppm/53 ms for ^15^N dimensions with D1 = 1 s and a constant time delay of T = 0.06 s. η_xy_ values were determined from a series of 9 relaxation delays: −0.05, −0.0375, −0.025, −0.0125, 0.0, 0.0125, 0.025, 0.0375, and 0.05. Carrier positions: ^1^H, H_2_O frequency (4.698 ppm); ^13^C, 95 ppm; ^15^N, 118.0 ppm. Mirror image linear prediction was used for constant-time ^15^N sampling, doubling resolution without introducing exponential decay artefacts. The errors in the η_xy_ experiment were defined by exponential decay fitting with cut-off at 5%.

An example illustrating the quality of the experimental data for the ^1^H-^15^N CSA/DD cross-correlation relaxation rates (η_xy_) acquired at 600 MHz using the pulse program shown in Figure 6 is provided in Supplementary File S2 and Figure S7.

### 4.5. Theoretical Simulations: AlphaFold3 as a Starting Point for Full Atomic Molecular Dynamic

We utilized the Google DeepMind AlphaFold3 service [111] to predict five Psr_Sp_ protein conformations (with ranking scores 0.88, 0.88, 0.87, 0.87, 0.87). These predicted conformations did not account for solution properties such as pH values, temperature, ionic strength, etc., so there is a need to align the simulations to our experimental conditions. For molecular dynamics (MD), the charge of the protein residues was calculated at pH = 6.8, with all histidine residues displaying neutral charges. The ionic strength was set to 138 mM, combining buffer and salt concentrations.

All MD simulations were performed using Gromacs version 2023.1 [112] with the all-atom force field charmm36-mar2019_cufix.ff, including a refinement of the Lennard-Jones [113,114] parameters (CUFIX) [115]. The protein was centred in a periodic cubic box (100 Å), with corresponding 31,060 TIP3P water molecules, and Na^+^ (95) and Cl^−^ (83) ions were added to emulate the ionic strength and achieve electro-neutrality, as the protein had the total charge of (−12). Long-range electrostatic and van der Waals interactions were considered with a 10 Å cut-off.

The predicted protein conformations underwent energy minimization to ensure a reasonable starting structure in terms of geometry and solvent orientation. Convergence was achieved at a maximum force of less than 1000 kJ/mol/nm for any atom. The potential energy was then used to select the best starting structure from the five AlphaFold3 predictions (potential energy value for each structure: (1) −1.596 × 10^6^ kJ/mol; (2) −1.562 × 10^6^ kJ/mol; (3) −1.575 × 10^6^ kJ/mol; (4) −1.608 × 10^6^ kJ/mol; (5) −1.593 × 10^6^ kJ/mol). The fourth structure with the lowest potential energy was chosen for MD simulations.

Equilibration was conducted in two phases: the NVT (number of particles (N), volume (V), and temperature (T) are constant) ensemble for 100 ps, where the temperature of the system should reach a plateau at the desired value and where temperature was set to 308 K, and the NPT (number of particles (N), pressure (P), and temperature (T) are constant) ensemble for 100 ps, until the system reached equilibrium, as indicated by a plateau in pressure and density values. A modified Berendsen-type (V-rescale) thermostat and a Parrinello–Rahman barostat were employed. Hydrogen-containing covalent bonds were constrained.

Following equilibration, MD simulations continued as a production run for 6000 ns under the same conditions. System stability was assessed using standard tools in Gromacs [112], monitoring temperature, pressure, energy, and periodicity. Visual inspection and RMSD and RMSF analysis were performed in the xmgrace program (Turner PJ. XMGRACE, Version 5.1.19. Center for Coastal and Land-Margin Research, Oregon Graduate Institute of Science and Technology, Beaverton, OR; 2005).

Cluster analysis were performed for trajectory regions 700–1200, 1750–2250, 2500–3000, and 4650–5150 ns. Then, 1000 trajectory frames with 500 ps steps for each trajectory region were partitioned into 4 arrays of 20 clusters each using the Gromacs algorithm.

### 4.6. Individual MD Trajectory Analyses with Back-Calculation of Theoretical ^15^N Relaxation Parameters

MD trajectory regions were analysed by back-calculation NMR spin-relaxation parameters, using a bootstrapping procedure to estimate parameter dispersion, as previously described [52]. Each 500 ns MD segment was selected to be several times longer than the maximum duration (7 × τ_c_ [26]) of the autocorrelation function C(t), to ensure proper averaging of C(t) values and effective application of the moving block bootstrap method [52]. For each MD segment, the analysis began by aligning all protein frames to the mean structure, using the heavy atoms of rigid backbone residues 131–152, 162–193, 204–265, 296–342, 351–366, and 383–392.

Backbone ^1^H-^15^N vector extraction and approximation of autocorrelation function *C*(*t*) to a multi-exponential decay, where

(3) $$C\left(t\right){=A}_{0}+\sum_{j=1}^{m}A_{j}e^{-t/\tau_{j}}$$

with the best-fit parameters *A*_0_, *A_j_*, *τ_j_* and the subsequent spectral density function J(*ω*) calculations, were utilized as previously described [52] with the aid of the “Mathematica” software package (Wolfram Research, Champaign, IL, USA) and the MD Analysis external library [mdanalysis.org]. Back-calculation of classical NMR ^15^N relaxation parameters *η_xy_*, *R*_1_, and *NOE* as a function of *J*(*ω*) were also performed as previously described [26,52], whereas ^1^H-^15^N CSA/DD cross-correlation contribution to transverse relaxation, denoted as follows:

(4) $$\eta_{xy}=\frac{1}{15}\left(\frac{\mu_{0}{h\gamma }_{H}\gamma_{N}\omega_{N}\Delta \sigma P_{2}\left(cos\left(\theta \right)\right)}{8\pi^{2}r_{NH}^{3}}\right)\left[4J\left(\omega_{H}-\omega_{N}\right)+3J\left(\omega_{N}\right)\right]$$

(5) $$R_{1}=\frac{1}{10}{\left(\frac{\mu_{0}{h\gamma }_{H}\gamma_{N}}{8\pi^{2}r_{\mathrm{NH}}^{3}}\right)}^{2}\left[J\left(\omega_{H}-\omega_{N}\right)+3J\left(\omega_{N}\right)+6J\left(\omega_{H}{+\omega }_{N}\right)\right]+\frac{2\omega_{N}^{2}{\Delta \sigma }^{2}}{15}J\left(\omega_{N}\right)$$

(6) $$NOE=1+\frac{\gamma_{H}^{3}\gamma_{N}}{10R_{1}}{\left(\frac{\mu_{0}h}{8\pi^{2}r_{NH}^{3}}\right)}^{2}\left[6J\left(\omega_{H}{+\omega }_{N}\right)-J\left(\omega_{H}-\omega_{N}\right)\right]$$

(7) $$R_{2}=\frac{1}{20}{\left(\frac{\mu_{0}{h\gamma }_{H}\gamma_{N}}{8\pi^{2}r_{\mathrm{NH}}^{3}}\right)}^{2}\left[4J\left(0\right)+3J\left(\omega_{N}\right)+J\left(\omega_{H}-\omega_{N}\right)+6J\left(\omega_{H}\right)+6J\left(\omega_{H}{+\omega }_{N}\right)\right]+\frac{\omega_{N}^{2}{\Delta \sigma }^{2}}{45}\left[4J\left(\omega_{0}\right)+3J\left(\omega_{N}\right)\right]$$

where the spectral density function was

(8) $$J\left(\omega \right)=\frac{A_{0}\tau_{c}}{1+{\left({\omega \tau }_{c}\right)}^{2}}+\sum_{j=1}^{m}\frac{A_{j}\tau_{j}\prime }{1+{\left({\omega \tau }_{j}\prime \right)}^{2}}$$

where $\tau_{j}{‘=\tau }_{c}\tau_{j}/\tau_{c}{+\tau }_{j}$; $\tau_{c}$ is the experimental rotation correlation time; ${µ}_{o}$ is the vacuum permeability; h is Planck’s constant; $Y_{H}$ and $Y_{N}$ are the gyromagnetic ratios of ^1^H and ^15^N, respectively; Δ*σ* is the chemical shift anisotropy (CSA) of ^15^N with Δ*σ* = −166 ± 9 ppm [116]; *r*_NH_ = 1.023 ± 0.006 Å [117]; *ω*_N_ and *ω*_H_ are the Larmor frequencies of ^15^N and ^1^H at 600 MHz, respectively; CSA tensor value with respect to the NH vector Δ*σP*_2_(Cos (θ)) = −145 ± 8 ppm, *θ* is the CSA/NH vector angle and *P*_2_ is the Legendre 2nd-degree polynomial [118]; *J*(*ω*) is the NH auto-correlation spectral density function.

### 4.7. Calculation of Chemical Shift Procedure

ShiftX2 v1.10 chemical shift prediction software [87] was used to predict chemical shifts from PDB structure sets of Psr_Sp_ protein. Averaged ^1^H, ^13^C, and ^15^N chemical shifts were calculated for 500 ns of free MD simulations, sampled every 0.5 ns, yielding 1000 structures per trajectory segment. The analysed trajectory regions (700–1200 ns, 1750–2250 ns, 2500–3000, and 4650–5150 ns) were simulated at 308 K and pH 6.8

### 4.8. Statistical Analysis

In this study, the primary limitation to applying the χ^2^ goodness-of-fit test was not the assumption of normality but rather the requirement that deviations between experimental and back-calculated relaxation parameters need to be independent and identically distributed [119]. Systematic, residue-correlated deviations violate this independence criterion, rendering χ^2^ statistics potentially misleading even when the residuals conform to a normal distribution. To account for such cases, we employed the nonparametric Mann–Whitney U test [86], which is robust for deviations from normality and does not rely on independence assumptions. Within this framework, a *p*-value ≤ 0.05 denotes rejection of the null hypothesis, indicating a statistically significant difference between the groups, whereas a *p*-value > 0.05 indicates that no statistically significant difference can be established.

## 5. Conclusions

Conformational heterogeneity is essential for protein function, necessitating approaches that move beyond static structures to capture dynamic ensembles. Traditional inverse modelling in biomolecular NMR spectroscopy typically estimates an average structure with angular fluctuations of NH vectors, whereas the segment-selected MD approach presented here provides a complete dynamic model of the molecular system under investigation.

Here, we introduce a novel method that combines free molecular dynamics (MD) simulations with NMR relaxation data. Our approach selects discrete segments of the MD trajectory with stable RMSD. These models contain substantially more detailed information, a set of conformations and their percentage contributions, capturing a complete motional description of backbone and side chains. The resulting 4D ensembles are validated directly against experimental relaxation parameters, ensuring consistency with physical observables. Importantly, the starting structure may be derived from any independent method (e.g., X-ray crystallography, cryo-EM, AlphaFold, NMR models, etc.), and any force field may be employed, as long as the final conformational ensemble is fitted to the experimental relaxation data.

While our approach is complementary to the well-established ABSURDer protocol for generating 4D ensembles, it offers a distinct advantage: conformational processes can be analysed over longer timescales defined by RMSD plateaus, rather than being restricted to short blocks of ~1τc as in ABSURDer. Shorter blocks reweighting risk disrupt causal relationships in the dynamics, whereas plateau-based selection preserves temporal continuity and enables the capture of larger, functionally relevant motions that may be inaccessible to block-based reweighting methods.

Successful implementation depends on high-quality ^15^N backbone relaxation measurements, though even partial backbone assignments may suffice. Additionally, the minimal segment length must exceed ~10–14 × τ_c_ to ensure statistical reliability.

Any 3D structural ensemble must either be used as a starting point for MD simulations or embedded within an MD trajectory to provide a sufficiently long time domain for calculating correlation functions. Consequently, the applicability of our method is restricted to continuous MD trajectories (i.e., 4D ensembles), rather than static 3D structure sets. Time is needed to build up correlation function of enough length so that it can be used to produce relaxation parameters.

The method also assumes that suitable candidate sub-trajectories exist. For intrinsically disordered proteins (IDPs), additional methodological developments and more advanced force fields (e.g., polarizable models) may be required.

We applied this approach to the P_srSp_ protein, using an AlphaFold-generated structure as the starting point and a set of backbone relaxation measurements. Our analysis revealed that only specific regions of the MD trajectory were consistent with experimental data, highlighting the critical importance of experimental validation for dynamic ensemble accuracy. Furthermore, we identified two flexible regions in P_srSp_ that may contribute to its catalytic function. The validated ensembles serve as a valuable foundation for future structural and functional studies, including the development of antibiotics targeting this protein.

Looking ahead, the method could be extended to a broader range of proteins, larger in size, including multidomain systems, and protein–ligand complexes. Incorporating additional experimental data such as ^13^C backbone and side-chain relaxations would further enhance model resolution. Integration with improved force fields optimized for both backbones and side-chains, including polarizable models and better representations of the environment, will also expand its applicability and enable more accurate representation of complex energy landscapes.

In addition, the development of automated fragmentation schemes will streamline ensemble selection, reduce user bias, and ultimately allow integration with machine-learning frameworks trained on large structural datasets.

Overall, this work demonstrates a resource-efficient and experimentally validated framework for characterizing conformational dynamics of folded proteins. By bridging MD simulations with relaxation-based validation, our approach expands the utility of integrative NMR–MD workflows and provides a foundation for future advances in ensemble modelling, structural biology, and drug discovery.

## Figures and Tables

**Table 1:** Root mean square deviation (RMSD *) between the most populated MD structures ** from trajectory segments (I)–(IV) and the three subunits (A, B, C) of the crystal structure of PsrSp.

X-Ray Subunit	(I) 700–1250 ns	(II) 1750–2250 ns	(III) 2500–3000 ns	(IV) 4650–5150 ns
A	1.14 Å (239 Cα)	1.74 Å (222 Cα)	1.68 Å (217 Cα)	1.815 Å (196 Cα)
B	1.02 Å (237 Cα)	1.55 Å (217 Cα)	1.645 Å (210 Cα)	1.778 Å (199 Cα)
C	1.13 Å (241 Cα)	1.52 Å (205 Cα)	1.65 Å (201 Cα)	2.015 Å (201 Cα)

* RMSD was calculated using Cα atoms with PyMOL, based on alignment of up to 290 residues. ** The structures used were the most populated clusters from each MD trajectory segment.

## Data Availability

Assignment data of PsrSp, with their R1, η_xy_, and NOE relaxation data experimentally obtained and back-calculated from free MD simulation together with calculated order parameters S2 and population values of conformational ensemble, were submitted to the BioMagResBank with accession code BMRB ID52556. Pulse sequence developed in this and our earlier study are available for Bruker Avance spectrometers https://github.com/lesovoydm. The structures of trajectory segment (I) have been deposited in the Protein Data Bank (PDB) (Entry ID: 9A9G).

## Supplementary Materials

The following supporting information can be downloaded at: https://www.mdpi.com/article/10.3390/ijms26188917/s1.
