# Supplementary material for: Accurate Protein Dynamic Conformational Ensembles: Combining AlphaFold, MD, and Amide 15N(1H) NMR Relaxation
Source: Int J Mol Sci. 2025 Sep 12;26(18):8917. doi: 10.3390/ijms26188917 (PMC12469643; doi:10.3390/ijms26188917)
Supplement: Supplementary file 1 [file ijms-26-08917-s001.zip › ijms-3814689-supplementary-update.pdf]

# Supporting Information

## Accurate Protein Dynamic Conformational Ensembles: Combining AlphaFold, MD and Amide $^{15}N(^{1}H)$ NMR Relaxation.

Dmitry Lesovoy[1*$], Konstantin Roshchin[1], Benedetta Maria Sala[2,3], Tatyana Sandalova[2,3], Adnane Achour[2,3], Tatiana Agback[5,6$], Peter Agback[6], Vladislav Orekhov[4,5]

[1]Shemyakin-Ovchinnikov Institute of Bioorganic Chemistry RAS, 117997 Moscow, Russia.

[2]Science for Life Laboratory, Department of Medicine, Solna, Karolinska Institute SE-17165 Solna.

[3]Division of Infectious Diseases, Karolinska University Hospital, SE-171 76 Stockholm, Sweden. adnane.achour@ki.se (A.A)

[4]Swedish NMR Centre, University of Gothenburg, Box 465, SE-40530 Gothenburg, Sweden.

[5]Department of Chemistry and Molecular Biology, University of Gothenburg, Box 465, SE-40530 tatiana.agback@slu.se (T.A.)

[6]Department of Molecular Sciences, Swedish University of Agricultural Sciences, PO Box 7015, SE-750 07 Uppsala, Sweden.

[$]Equal contribution

*Corresponding authors: lesovoydm@gmail.com (D.L.), vladislav.orekhov@nmr.gu.se (V.A.)

## Supplementary File S1

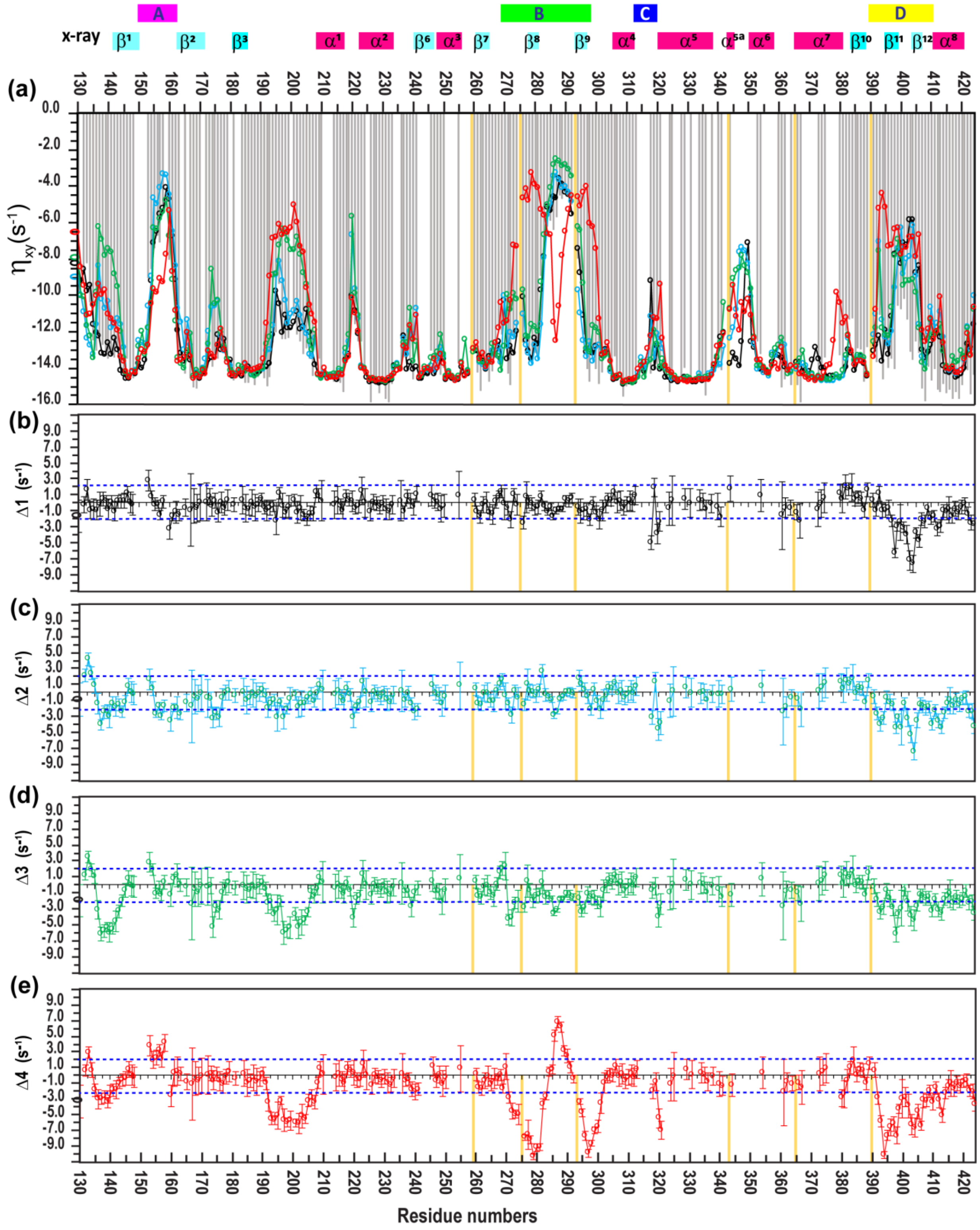


**Figure S1. $P_{srSp}$ amide backbone $^{15}$N(H) dynamic parameters obtained on a 600MHz spectrometer**. Panel (**a**) presents the experimentally obtained $^{1}$H-$^{15}$N CSA/DD cross-correlation relaxation ($\eta_{xy}$) data, shown as light grey solid bars. The theoretically recalculated from $\eta_{xy}$ values from MD trajectory data are displayed as solid lines in black, blue, green and red, corresponding

to ensembles of segments (I) 700-1200ns, (II) 1750-2250ns, (III) 2500-3000ns and (IV) 4650-5150 ns, respectively. Panels (b)-(e) display the differences between experimental and calculated $\eta_{xy}$ values for ensembles (I)-(IV), respectively, labelled on the axis as Δ1-Δ4. Error bars represent the square root of the sum of the squared experimental and theoretical errors. Theoretical errors were estimated using bootstrap analysis, as described in the Methods section. The one-sigma (1σ) level, derived from the Δ1–Δ4 datasets, is indicated by a dotted line in Panels (b)–(e). Long black and yellow markers indicate the positions of praline residues. The secondary structural elements of $P_{srSp}$ are shown at the top of the panels, based on the crystal structure of $P_{srSp}$ proteins previously determined by us[75]. A-D loop regions of $P_{srSp}$ are shown in pink, green, dark blue and yellow, respectively.

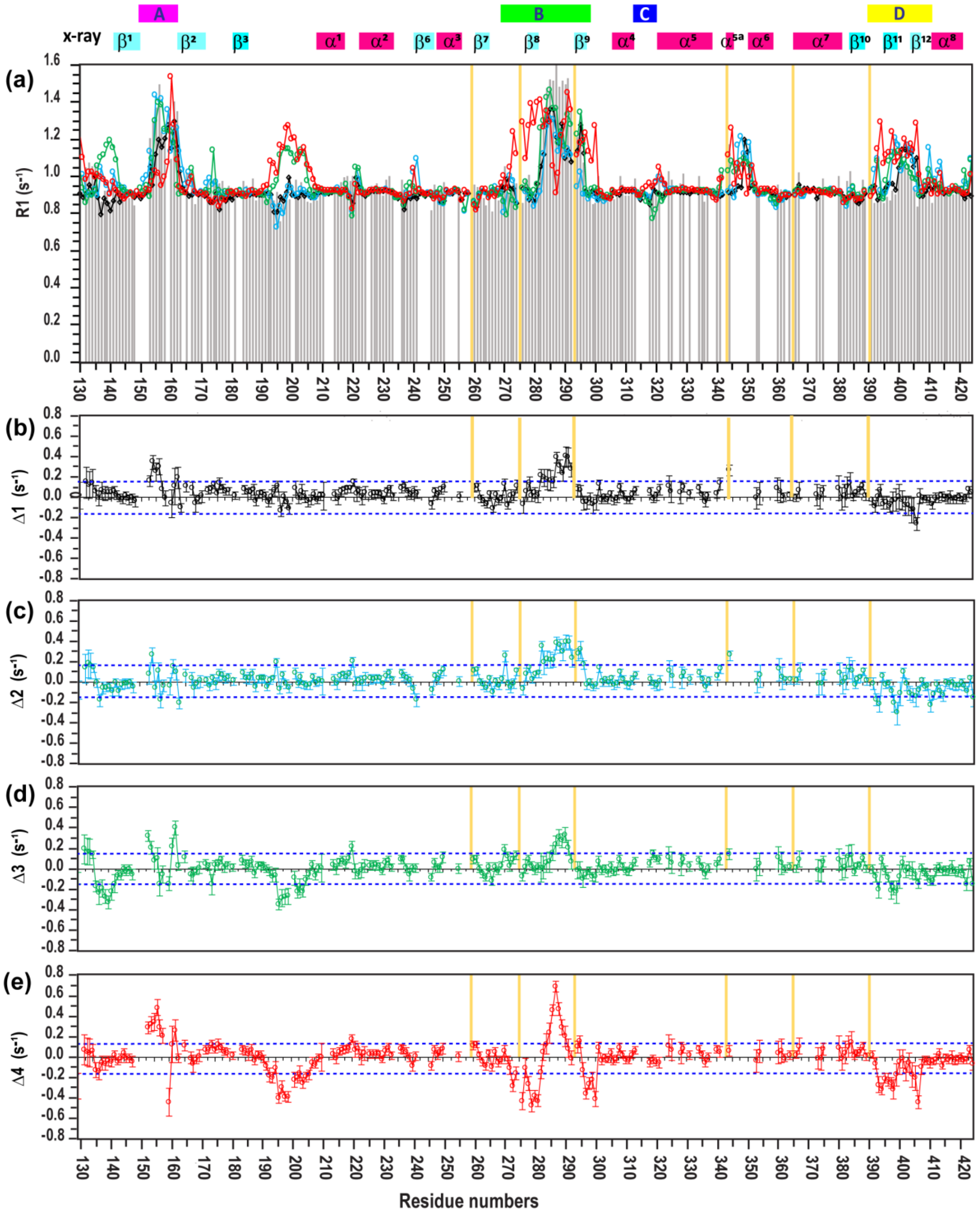


**Figure S2. $P_{srSp}$ amide backbone $^{15}N(H)$ dynamic parameters obtained on a 600MHz spectrometer**. Panel (**a**) presents the experimentally obtained $^{15}N$ longitude relaxation ($R_1$) data, shown as light grey solid bars. The theoretically recalculated from $R_1$ values from MD trajectory data are displayed as solid lines in black, blue, green and red, corresponding to ensembles of

segments (I) 700-1200ns, (II) 1750-2250ns, (III) 2500-3000ns and (IV) 4650-5150 ns, respectively. Panels (b)-(e) display the differences between experimental and calculated $R_1$ values for ensembles (I)-(IV), respectively, labelled on the axis as Δ1-Δ4. Error bars represent the square root of the sum of the squared experimental and theoretical errors. Theoretical errors were estimated using bootstrap analysis, as described in the Methods section. The one-sigma (σ) level, derived from the Δ1–Δ4 datasets, is indicated by a dotted line in Panels (b)–(e). Long black and yellow markers indicate the positions of praline residues. The secondary structural elements of $P_{srSp}$ are shown at the top of the panels, based on the crystal structure of $P_{srSp}$ proteins previously determined by us[75]. A-D loop regions of $P_{srSp}$ are shown in pink, green, dark blue and yellow, respectively.

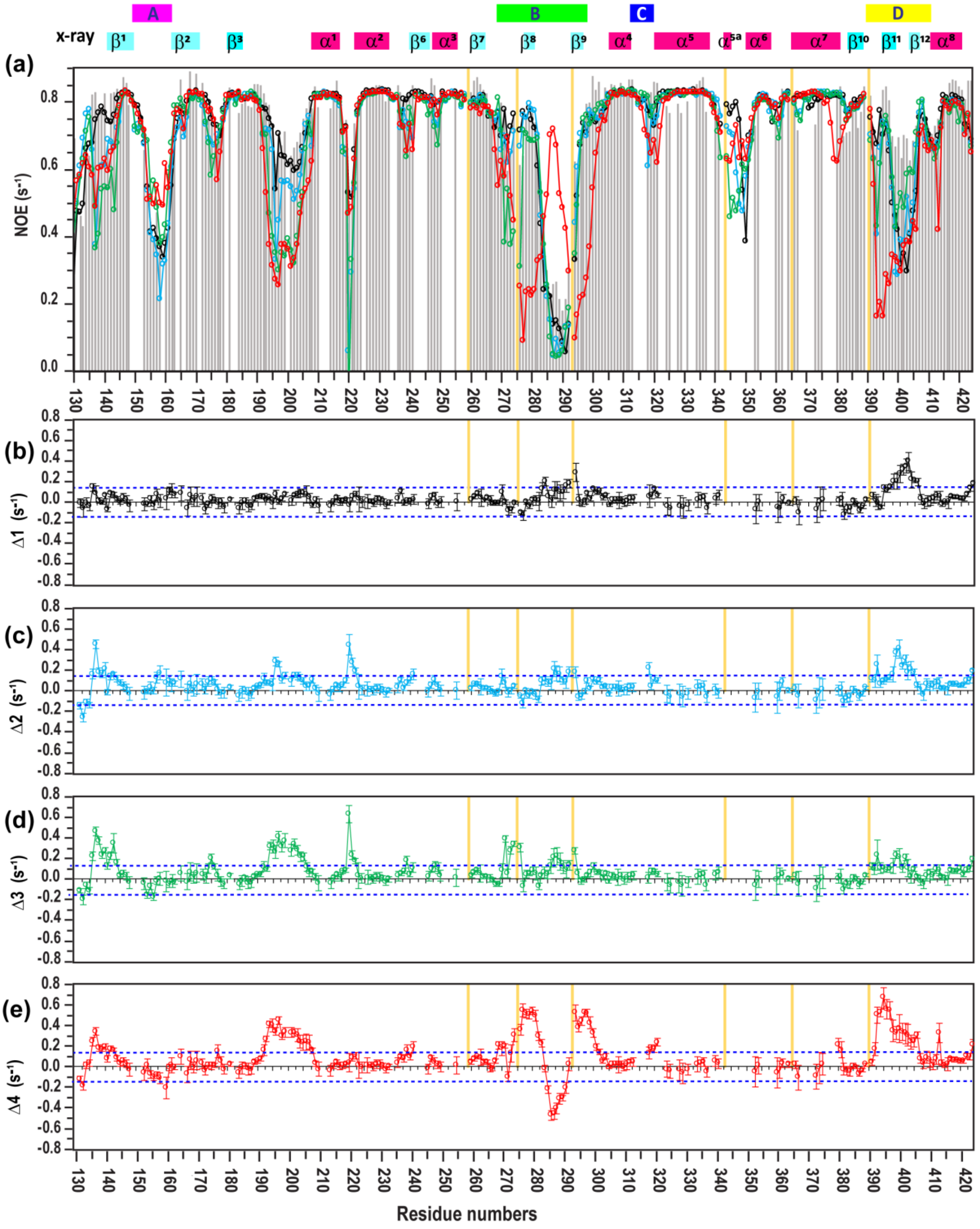


**Figure S3.** $P_{srSp}$ **amide backbone** $^{15}N(H)$ **dynamic parameters obtained on a 600MHz spectrometer**.

Panel (**a**) presents the experimentally obtained $^{1}H$-$^{15}N$ heteronuclear relaxation (NOE) data, shown as light grey solid bars. The theoretically recalculated from NOE values from MD trajectory data are displayed as solid lines in black, blue, green and red, corresponding to ensembles of segments (I) 700-1200ns, (II) 1750-2250ns, (III) 2500-3000ns and (IV) 4650-5150 ns, respectively. Panels (b)-(e) display the differences between experimental and calculated NOE values for ensembles (I)-(IV), respectively, labelled on the axis as Δ1-Δ4. Error bars represent the square root of the sum of the squared experimental and theoretical errors. Theoretical errors were estimated using bootstrap analysis, as described in the Methods section. The one-sigma (σ) level, derived from the Δ1–Δ4 datasets, is indicated by a dotted line in Panels (b)–(e). Long black and yellow markers indicate the positions of praline residues. The secondary structural elements of $P_{srSp}$ are shown at the top of the panels, based on the crystal structure of $P_{srSp}$ proteins previously determined by us[75]. A-D loop regions of $P_{srSp}$ are shown in pink, green, dark blue and yellow, respectively.

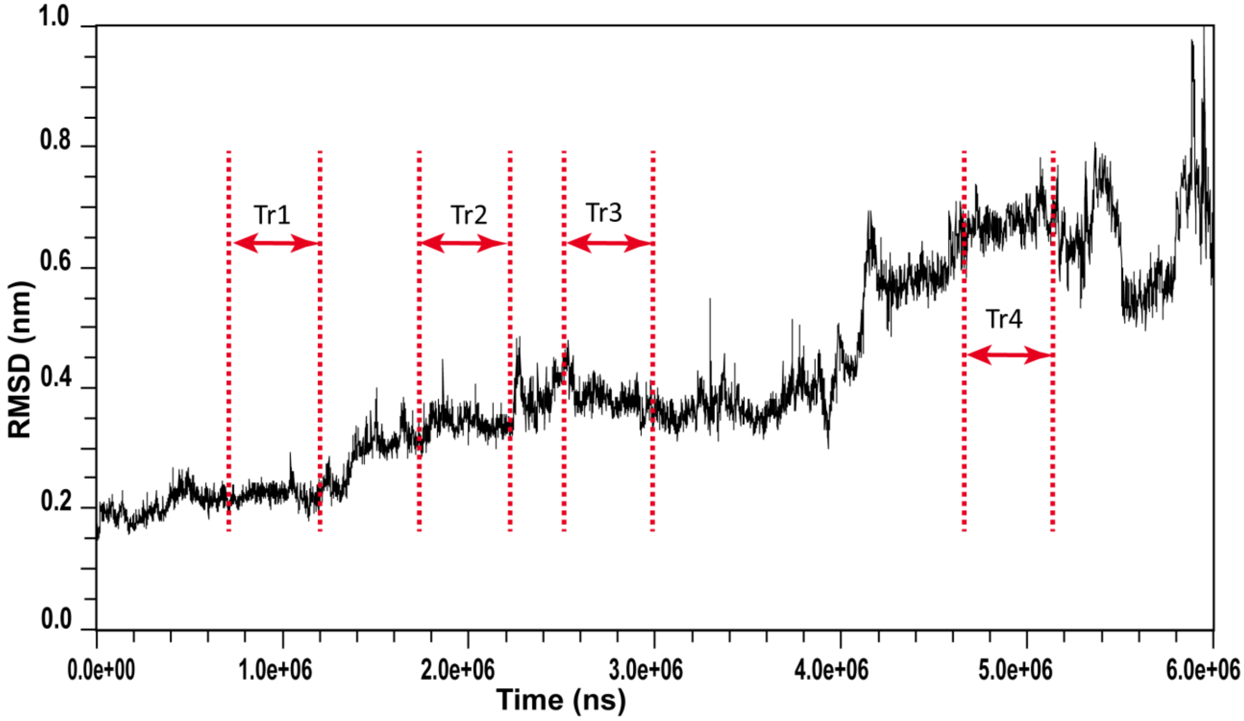


**Figure S4. RMSD along the MD trajectory**.

RMSD vs the initial structure for the backbone heavy atoms of $P_{srSp}$ obtained during 6us MD trajectory. The starting structure was obtained from AF of $P_{srSp}$. Four trajectory intervals (I) 700-1200ns, (II) 1750-2250ns, (III) 2500-3000ns and (IV) 4650-5150ns with length 500ns used for back calculations of the relaxation $R_1$, $\eta_{xy}$ and NOE parameters of protein are depicted by red arrows**.**

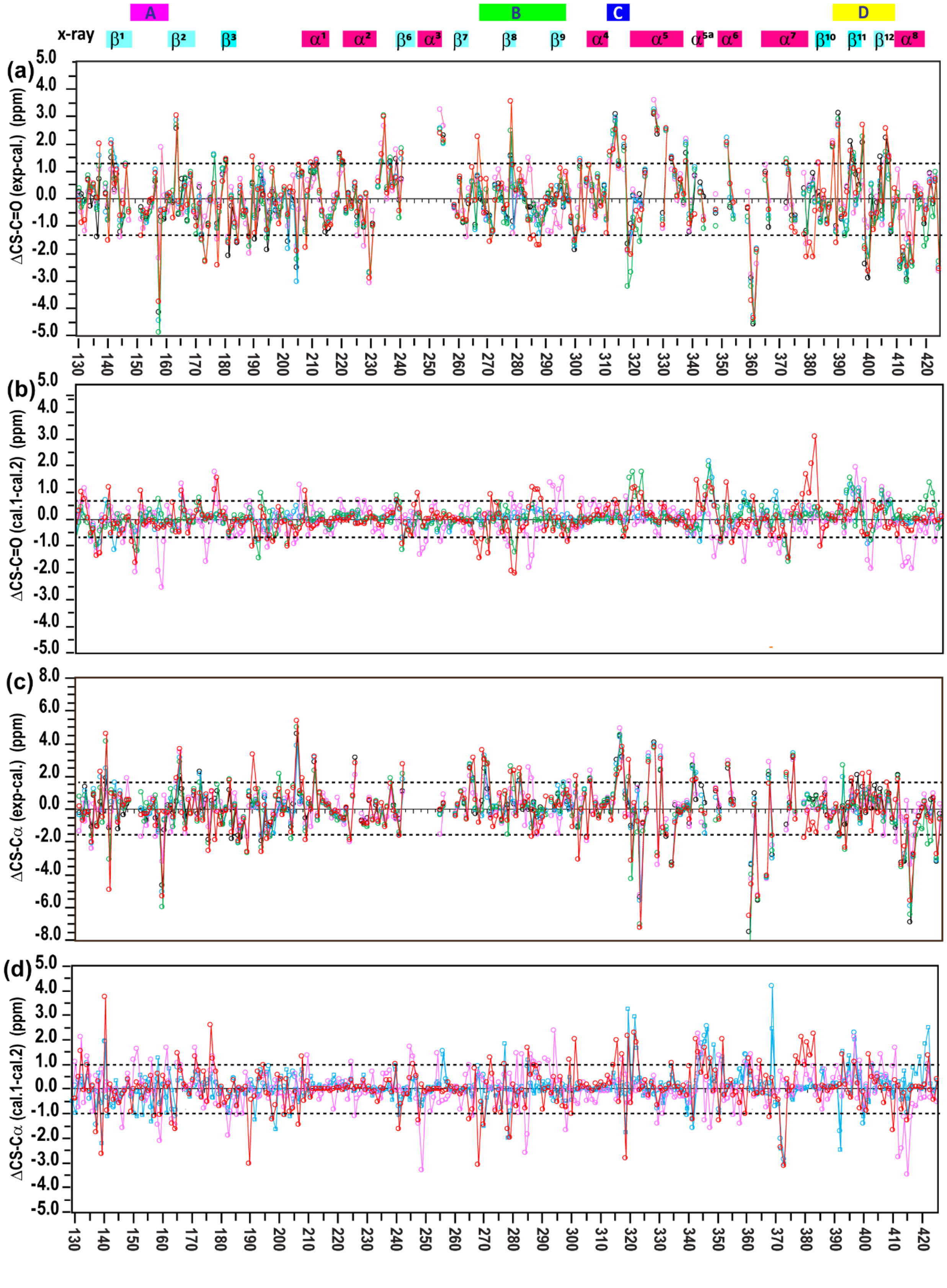

A
B
C
D
x-ray
β1 β2 β3 α1 α2 β6 α3 β7 β8 β9 α4 α5 α5a α6 α7 β10 β11 β12 α8
(a)
ΔCS-C=O (exp-cal.) (ppm)
(b)
ΔCS-C=O (cal.1-cal.2) (ppm)
(c)
ΔCS-Cα (exp-cal.) (ppm)
(d)
ΔCS-Cα (cal.1-cal.2) (ppm)

**Figure S5. $P_{srSp}$ experimentally obtained and calculated from MD ensembles Cα and C=O chemical shifts.**

Panels (a) and (c) show the differences between the experimentally obtained C=O and Cα chemical shifts and those recalculated from conformational ensembles (I)–(IV), labelled on the axis as ΔC=O (exp–cal.) and ΔCα (exp–cal.), respectively. Chemical shift values from the MD trajectory data are shown as solid lines in black, blue, green, and red, corresponding to the ensembles from segments (I) 700–1200 ns, (II) 1750–2250 ns, (III) 2500–3000 ns, and (IV) 4650–5150 ns, respectively. Chemical shift values recalculated from the X-ray structure are shown as solid pink lines. Panels (b) and (d) display the differences in chemical shifts between calculated ensembles (I)–(IV), labelled on the axis as ΔC=O (cal.1–cal.2) and ΔCα (cal.1–cal.2), respectively. The chemical shift differences between MD trajectories are shown as solid lines in blue, green, and red, corresponding to the differences between ensembles 700–1200 ns vs. 1750–2250 ns, 700–1200 ns vs. 2500–3000 ns, and 700–1200 ns vs. 4650–5150 ns, respectively. The differences between MD trajectory segment (I) and the X-ray structure are shown as solid pink lines. The one-sigma (σ) level, derived from the difference data, is indicated by dashed lines. The secondary structural elements of $P_{srSp}$, based on the crystal structure, are shown at the top of the panels. A-D loop regions of $Psr_{Sp}$ are shown in pink, green, dark blue and yellow, respectively.

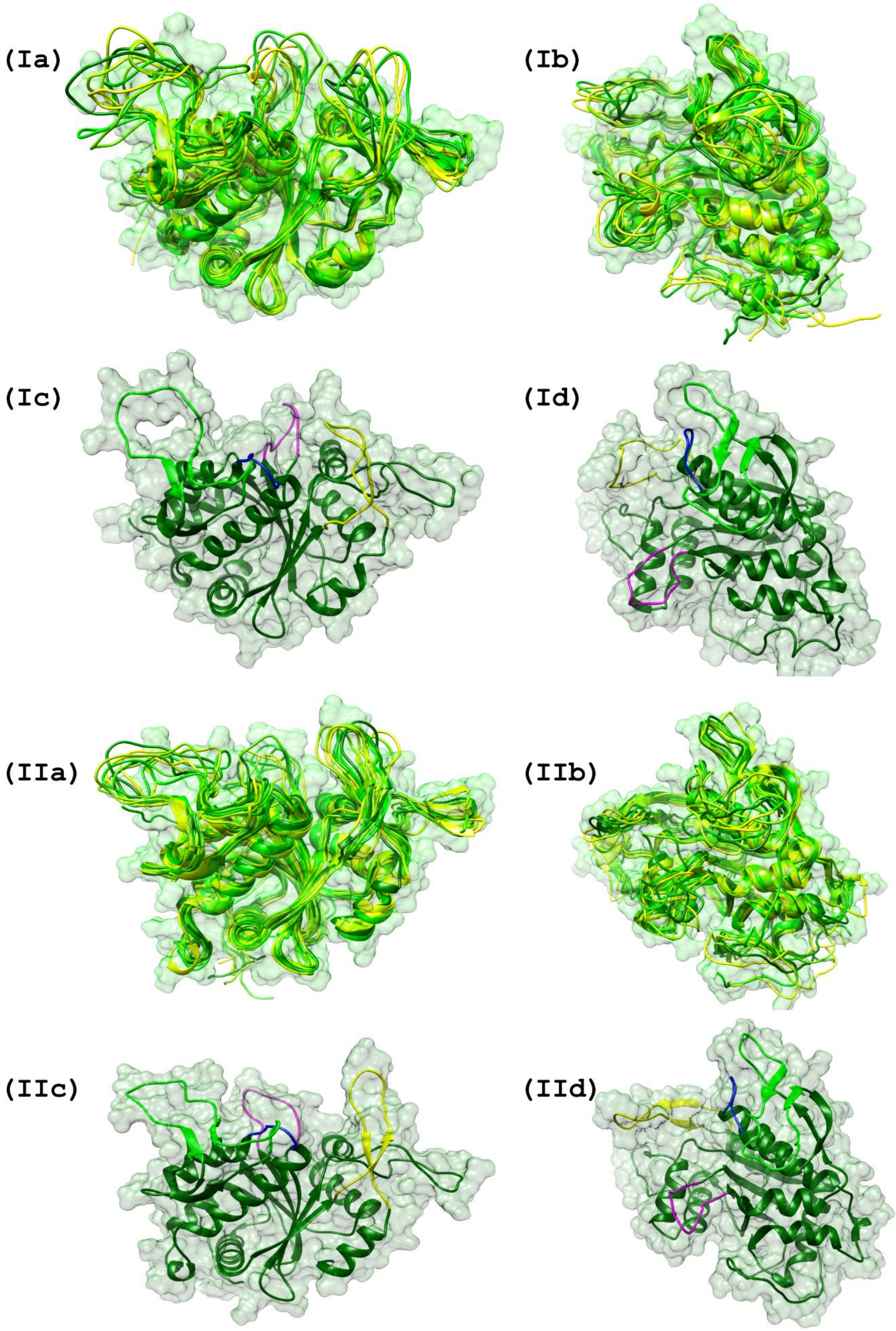
(Ia)
(Ib)
(Ic)
(Id)
(IIa)
(IIb)
(IIc)
(IId)

**Figure S6. Two Conformational Ensembles (I-II) of $P_{srSp}$ Obtained through 6 µs Long MD Free-Restraint Simulations and Verified by NMR.**

Panels (Ia-d) and (IIa-d) depict ribbon representations of the domain structures of the two ensembles, each comprising 10 models of $P_{srSp}$. These models were obtained through clustering analysis of trajectory cluster (I) (700–1200 ns) and trajectory cluster (II) (1750–2250 ns), respectively. In panels (**Ia,b**) and (**IIa,b**) the globular $P_{srSp}$ domain, spanning residues 130–424, is color-coded according to **Table S2**, with the clusters ranging from highest to lowest populations represented by dark green to yellow, respectively. Panels (**Ic,d**) and (**IIc,d**) illustrate the first structures of the most populated clusters from trajectory segments (I) and (II), respectively. These structures are displayed in dark green, except for the residues associated with the A, B, C, and D substrate-binding sites of $P_{srSp}$, which are highlighted in hot pink, green, dark blue, and yellow, respectively. The structures shown on the right (**Ib,d**) and (**IIb,d**) represent 180-degree rotations of the corresponding structures in panels (**Ia,c**) and (**IIa,c**). The surfaces corresponding to the van der Waals radii of each heteroatom in the $P_{srSp}$ protein are rendered in transparent white-grey.

**Table S1** Experimentally obtained relaxation parameters $R_1$, $\eta_{xy}$ and NOE with their errors (σ) of the $P_{srSp}$ protein.

| N residue | Peak name | $R_1$ ($s^{-1}$) | σ ($R_1$) | NOE | σ (NOE) | $\eta_{xy}$ ($s^{-1}$) | σ ($\eta_{xy}$) |
|---|---|---|---|---|---|---|---|
| 130 | 1Gly | 0.925 | 0.046 | 0.690 | 0.078 | -9.92 | 1.80 |
| 131 | 2Glu | | | | | | |
| 132 | 3Val | 1.049 | 0.133 | 0.463 | 0.019 | -8.83 | 0.44 |
| 133 | 4Glu | 1.076 | 0.128 | 0.430 | 0.039 | -8.18 | 0.41 |
| 134 | 5Val | 1.056 | 0.118 | 0.654 | 0.033 | -10.38 | 0.52 |
| 135 | 6Phe | 1.061 | 0.053 | 0.639 | 0.032 | -12.44 | 0.62 |
| 136 | 7Asn | 0.882 | 0.044 | 0.822 | 0.041 | -13.00 | 1.00 |
| 137 | 8Gly | 0.862 | 0.043 | 0.828 | 0.041 | -12.40 | 0.62 |
| 138 | 9Gln | 0.921 | 0.046 | 0.782 | 0.039 | -12.70 | 0.64 |
| 139 | 10Asp | 0.892 | 0.045 | 0.776 | 0.039 | -13.16 | 0.66 |
| 140 | 11Thr | 0.864 | 0.043 | 0.827 | 0.041 | -13.43 | 1.00 |
| 141 | 12Arg | 0.926 | 0.046 | 0.672 | 0.034 | -13.20 | 0.66 |
| 142 | 13Asp | 0.939 | 0.047 | 0.825 | 0.041 | -13.40 | 1.00 |
| 143 | 14Gly | 0.858 | 0.043 | 0.830 | 0.041 | -12.87 | 0.64 |
| 144# | 15Val | 0.874 | 0.044 | 0.840 | 0.042 | -13.99 | 0.70 |
| 145# | 16Asn | 0.917 | 0.046 | 0.819 | 0.041 | -14.09 | 0.70 |
| 146# | 17Ile | 0.883 | 0.044 | 0.873 | 0.044 | -13.45 | 0.67 |
| 147# | 18Leu | 0.901 | 0.045 | 0.857 | 0.043 | -14.50 | 1.00 |
| 148# | 19Ile | 0.844 | 0.042 | 0.792 | 0.059 | -14.50 | 1.00 |
| 149 | 20Met | | | | | | |
| 150 | 21Gly | | | | | | |
| 151 | 22Thr | | | | | | |

| | | | | | | | |
|---|---|---|---|---|---|---|---|
| 152 | 23Asp | | | | | | |
| 153 | 24Gly | 1.208 | 0.060 | 0.655 | 0.060 | -9.00 | 1.00 |
| 154 | 25Arg | 1.296 | 0.065 | 0.529 | 0.026 | -8.49 | 0.42 |
| 155 | 26Ile | 1.370 | 0.068 | 0.444 | 0.026 | -7.38 | 0.37 |
| 156 | 27Gly | 1.496 | 0.075 | 0.395 | 0.023 | -6.70 | 0.33 |
| 157 | 28Gln | 1.236 | 0.062 | 0.470 | 0.026 | -7.08 | 0.35 |
| 158 | 29Asn | 1.196 | 0.060 | 0.386 | 0.053 | -5.08 | 0.70 |
| 159 | 30Ser | | | | | | |
| 160 | 31Val | 1.090 | 0.055 | 0.411 | 0.050 | -8.00 | 1.30 |
| 161 | 32Glu | 1.401 | 0.070 | 0.536 | 0.027 | -8.44 | 0.42 |
| 162 | 33Thr | 1.349 | 0.067 | 0.660 | 0.059 | -10.30 | 1.26 |
| 163 | 34Arg | 0.923 | 0.046 | 0.761 | 0.038 | -13.48 | 0.67 |
| 164 | 35Thr | | | | | | |
| 165# | 36Asp | 1.022 | 0.051 | 0.865 | 0.075 | -14.28 | 0.71 |
| 166 | 37Ser | | | | | | |
| 167# | 38Ile | 0.986 | 0.049 | 0.741 | 0.079 | -14.50 | 2.50 |
| 168# | 39Met | 0.850 | 0.076 | 0.889 | 0.087 | -15.01 | 2.00 |
| 169# | 40Val | 0.883 | 0.044 | 0.821 | 0.052 | -15.07 | 1.50 |
| 170# | 41Leu | 0.919 | 0.047 | 0.885 | 0.090 | -14.57 | 2.11 |
| 171 | 42Asn | | | | | | |
| 172 | 43Val | 0.970 | 0.049 | 0.855 | 0.049 | -14.25 | 2.29 |
| 173 | 44Gly | 0.940 | 0.047 | 0.751 | 0.046 | -12.85 | 0.64 |
| 174 | 45Gly | 0.983 | 0.049 | 0.782 | 0.039 | -13.88 | 1.00 |
| 175 | 46Ser | 0.947 | 0.047 | 0.785 | 0.039 | -13.20 | 0.66 |
| 176 | 47Asp | 0.959 | 0.048 | 0.757 | 0.038 | -13.45 | 1.00 |
| 177 | 48Lys | 0.972 | 0.049 | 0.716 | 0.050 | -13.00 | 1.00 |
| 178 | 49Lys | 0.922 | 0.046 | 0.701 | 0.038 | -13.14 | 0.66 |
| 179 | 50Met | 0.948 | 0.047 | 0.724 | 0.046 | -13.24 | 1.00 |
| 180 | 51Lys | | | | | | |
| 181# | 52Leu | 0.918 | 0.046 | 0.843 | 0.023 | -14.67 | 0.73 |
| 182 | 53Val | | | | | | |
| 183 | 54Ser | | | | | | |
| 184# | 55Phe | 0.977 | 0.049 | 0.768 | 0.050 | -14.08 | 1.50 |
| 185# | 56Met | 0.950 | 0.048 | 0.794 | 0.055 | -14.75 | 1.50 |
| 186# | 57Arg | 0.940 | 0.047 | 0.823 | 0.041 | -14.76 | 0.74 |
| 187# | 58Asp | 0.988 | 0.049 | 0.787 | 0.059 | -14.45 | 1.48 |
| 188# | 59Asn | 0.867 | 0.043 | 0.783 | 0.039 | -14.90 | 1.50 |
| 189# | 60Leu | 0.932 | 0.047 | 0.851 | 0.043 | -14.48 | 0.72 |
| 190# | 61Val | 0.936 | 0.047 | 0.854 | 0.043 | -13.76 | 0.69 |
| 191# | 62Tyr | 0.923 | 0.046 | 0.850 | 0.042 | -14.53 | 0.73 |
| 192 | 63Ile | 0.857 | 0.043 | 0.824 | 0.044 | -15.00 | 1.00 |
| 193 | 64Asp | 0.902 | 0.045 | 0.798 | 0.040 | -14.44 | 0.72 |
| 194 | 65Gly | 0.869 | 0.043 | 0.792 | 0.040 | -12.60 | 0.63 |
| 195 | 66Tyr | 0.915 | 0.046 | 0.717 | 0.050 | -12.00 | 1.70 |
| 196 | 67Ser | 0.757 | 0.038 | 0.617 | 0.026 | -9.33 | 0.47 |
| 197 | 68Gln | 0.864 | 0.043 | 0.708 | 0.035 | -12.40 | 1.27 |
| 198 | 69Val | 0.868 | 0.043 | 0.669 | 0.033 | -12.35 | 0.62 |
| 199 | 70Ile | 0.879 | 0.044 | 0.711 | 0.036 | -12.31 | 1.16 |
| 200 | 71Asn | | | | | | |
| 201 | 72Gly | 0.980 | 0.049 | 0.641 | 0.040 | -11.13 | 0.56 |
| 202 | 73Arg | 0.882 | 0.044 | 0.665 | 0.033 | -12.13 | 1.16 |
| 203 | 74Lys | 0.967 | 0.048 | 0.680 | 0.034 | -12.50 | 1.40 |

| | | | | | | | |
|---|---|---|---|---|---|---|---|
| 204 | 75Gln | 0.899 | 0.045 | 0.710 | 0.036 | -13.23 | 0.66 |
| 205 | 76Thr | 0.866 | 0.043 | 0.750 | 0.038 | -12.86 | 0.64 |
| 206 | 77Asp | 0.859 | 0.043 | 0.781 | 0.040 | -13.46 | 0.67 |
| 207 | 78Asn | 0.929 | 0.046 | 0.800 | 0.040 | -14.42 | 0.72 |
| 208[#] | 79Lys | 0.869 | 0.043 | 0.779 | 0.039 | -12.44 | 0.62 |
| 209[#] | 80Leu | 0.926 | 0.046 | 0.844 | 0.042 | -13.48 | 0.67 |
| 210[#] | 81Asn | 0.926 | 0.130 | 0.784 | 0.070 | -14.23 | 2.40 |
| 211 | 82Val | | | | | | |
| 212 | 83Ala | | | | | | |
| 213 | 84Tyr | | | | | | |
| 214[#] | 85Glu | 0.949 | 0.047 | 0.780 | 0.052 | -14.74 | 1.50 |
| 215[#] | 86Leu | 0.908 | 0.045 | 0.826 | 0.041 | -14.32 | 1.47 |
| 216[#] | 87Gly | 0.965 | 0.048 | 0.863 | 0.043 | -14.80 | 0.74 |
| 217[#] | 88Glu | 0.992 | 0.050 | 0.816 | 0.041 | -15.30 | 0.76 |
| 218 | 89Gln | 1.002 | 0.050 | 0.723 | 0.036 | -12.74 | 0.64 |
| 219 | 90Glu | 0.977 | 0.049 | 0.745 | 0.037 | -12.84 | 0.64 |
| 220 | 91Gly | 1.001 | 0.050 | 0.506 | 0.055 | -9.82 | 1.15 |
| 221 | 92Gln | 1.025 | 0.051 | 0.569 | 0.028 | -11.54 | 0.58 |
| 222 | 93Lys | 0.991 | 0.050 | 0.735 | 0.037 | -12.53 | 0.63 |
| 223[#] | 94Gly | 0.908 | 0.065 | 0.800 | 0.124 | -12.52 | 1.54 |
| 224[#] | 95Ala | 0.937 | 0.055 | 0.800 | 0.048 | -14.03 | 1.66 |
| 225 | 96Glu | | | | | | |
| 226[#] | 97Met | 0.950 | 0.048 | 0.840 | 0.070 | -15.83 | 0.79 |
| 227[#] | 98Val | 0.950 | 0.048 | 0.834 | 0.042 | -14.59 | 0.73 |
| 228[#] | 99Arg | 0.961 | 0.048 | 0.828 | 0.041 | -14.93 | 0.75 |
| 229[#] | 100Gln | 0.945 | 0.047 | 0.850 | 0.043 | -15.32 | 0.77 |
| 230[#] | 101Val | 0.896 | 0.045 | 0.850 | 0.043 | -14.87 | 0.74 |
| 231[#] | 102Leu | 0.983 | 0.049 | 0.843 | 0.042 | -15.66 | 1.00 |
| 232[#] | 103Lys | 1.001 | 0.050 | 0.801 | 0.040 | -15.82 | 0.79 |
| 233[#] | 104Asp | 0.927 | 0.046 | 0.826 | 0.041 | -14.86 | 0.74 |
| 234 | 105Asn | | | | | | |
| 235 | 106Phe | | | | | | |
| 236 | 107Asp | 0.979 | 0.049 | 0.851 | 0.047 | -13.83 | 2.42 |
| 237 | 108Leu | 0.930 | 0.046 | 0.829 | 0.041 | -13.40 | 0.67 |
| 238 | 109Asp | 0.944 | 0.047 | 0.801 | 0.040 | -13.38 | 0.67 |
| 239 | 110Ile | 0.902 | 0.045 | 0.818 | 0.041 | -13.49 | 0.67 |
| 240 | 111Lys | 0.949 | 0.047 | 0.825 | 0.041 | -14.59 | 0.73 |
| 241 | 112Tyr | 0.918 | 0.046 | 0.850 | 0.045 | -12.89 | 0.64 |
| 242 | 113Tyr | | | | | | |
| 243 | 114Ala | | | | | | |
| 244 | 115Leu | | | | | | |
| 245 | 116Val | | | | | | |
| 246[#] | 117Asp | 0.819 | 0.041 | 0.789 | 0.049 | -13.02 | 1.00 |
| 247[#] | 118Phe | 0.954 | 0.048 | 0.870 | 0.044 | -14.17 | 0.71 |
| 248[#] | 119Gln | 0.916 | 0.046 | 0.850 | 0.042 | -13.94 | 0.70 |
| 249[#] | 120Ala | 0.932 | 0.047 | 0.780 | 0.039 | -14.31 | 1.44 |
| 250[#] | 121Phe | 0.966 | 0.048 | 0.800 | 0.050 | -13.97 | 1.23 |
| 251 | 122Ala | | | | | | |
| 252 | 123Thr | | | | | | |
| 253 | 124Ala | | | | | | |
| 254 | 125 Ile | | | | | | |
| 255 | 126Asp | 0.923 | 0.046 | 0.830 | 0.084 | -13.70 | 2.83 |

| | | | | | | | |
|---|---|---|---|---|---|---|---|
| 256 | 127Thr | | | | | | |
| 257 | 128Leu | | | | | | |
| 258 | 129Phe | | | | | | |
| 259 | 130Pro | | | | | | |
| 260 | 131Asp | 0.956 | 0.048 | 0.827 | 0.041 | -12.74 | 0.64 |
| 261 | 132Gly | 0.939 | 0.047 | 0.864 | 0.043 | -14.21 | 0.71 |
| 262 | 133Val | 0.898 | 0.045 | 0.872 | 0.044 | -15.23 | 0.76 |
| 263 | 134Thr | 0.883 | 0.044 | 0.853 | 0.080 | -13.91 | 0.70 |
| 264 | 135Ile | 0.844 | 0.042 | 0.834 | 0.042 | -14.08 | 1.00 |
| 265 | 136Asp | 0.921 | 0.046 | 0.826 | 0.041 | -14.69 | 0.73 |
| 266 | 137Ala | 0.813 | 0.041 | 0.777 | 0.039 | -14.14 | 1.00 |
| 267 | 138Gln | 0.941 | 0.047 | 0.750 | 0.037 | -13.17 | 0.66 |
| 268 | 139Phe | 0.872 | 0.044 | 0.714 | 0.036 | -11.78 | 0.59 |
| 269 | 140Ser | 0.932 | 0.047 | 0.759 | 0.038 | -12.10 | 0.60 |
| 270 | 141Thr | 1.054 | 0.053 | 0.799 | 0.066 | -10.43 | 1.55 |
| 271 | 142Leu | 0.910 | 0.046 | 0.779 | 0.039 | -14.26 | 0.71 |
| 272 | 143Asn | 0.946 | 0.047 | 0.596 | 0.038 | -14.94 | 0.75 |
| 273 | 144Gly | 0.958 | 0.048 | 0.652 | 0.033 | -12.21 | 0.61 |
| 274 | 145Arg | 0.960 | 0.048 | 0.756 | 0.038 | -12.24 | 1.17 |
| 275 | 146Pro | | | | | | |
| 276 | 147Leu | 0.859 | 0.043 | 0.614 | 0.014 | -12.57 | 0.63 |
| 277 | 148Thr | 0.984 | 0.052 | 0.641 | 0.047 | -11.80 | 1.82 |
| 278 | 149Glu | 1.011 | 0.051 | 0.752 | 0.038 | -12.95 | 1.28 |
| 279 | 150Ala | 0.917 | 0.046 | 0.742 | 0.037 | -13.68 | 1.24 |
| 280 | 151Thr | 0.907 | 0.045 | 0.766 | 0.038 | -13.14 | 0.66 |
| 281 | 152Val | 0.969 | 0.048 | 0.740 | 0.043 | -13.21 | 0.66 |
| 282 | 153Gly | 1.265 | 0.063 | 0.620 | 0.031 | -8.94 | 0.45 |
| 283 | 154Asp | 1.308 | 0.065 | 0.564 | 0.028 | -8.27 | 0.41 |
| 284 | 155Asp | 1.479 | 0.074 | 0.440 | 0.022 | -6.21 | 0.31 |
| 285 | 156Leu | 1.525 | 0.076 | 0.301 | 0.015 | -5.86 | 0.29 |
| 286 | 157Tyr | 1.513 | 0.076 | 0.229 | 0.050 | -6.36 | 0.32 |
| 287 | 158Ala | 1.598 | 0.080 | 0.259 | 0.050 | -5.70 | 0.29 |
| 288 | 159Thr | 1.485 | 0.074 | 0.266 | 0.050 | -4.45 | 0.22 |
| 289 | 160Glu | 1.514 | 0.076 | 0.216 | 0.050 | -4.68 | 0.23 |
| 290 | 161Thr | 1.493 | 0.075 | 0.181 | 0.016 | -3.96 | 0.35 |
| 291 | 162Glu | 1.531 | 0.077 | 0.214 | 0.050 | -4.30 | 0.53 |
| 292 | 163Ser | 1.351 | 0.068 | 0.321 | 0.016 | -5.00 | 0.30 |
| 293 | 164Pro | | | | | | |
| 294 | 165Thr | 1.212 | 0.061 | 0.622 | 0.031 | -7.97 | 0.40 |
| 295 | 166Gln | 1.334 | 0.067 | 0.545 | 0.027 | -9.85 | 0.49 |
| 296 | 167Thr | 1.072 | 0.054 | 0.660 | 0.033 | -12.14 | 0.61 |
| 297 | 168Ile | 0.870 | 0.044 | 0.745 | 0.037 | -13.99 | 0.70 |
| 298 | 169Lys | 0.876 | 0.044 | 0.795 | 0.040 | -14.72 | 0.74 |
| 299 | 170Val | 0.850 | 0.042 | 0.797 | 0.040 | -13.27 | 0.66 |
| 300 | 171Gly | 0.857 | 0.043 | 0.875 | 0.044 | -14.02 | 0.70 |
| 301 | 172Lys | 1.002 | 0.050 | 0.835 | 0.042 | -14.84 | 1.00 |
| 302 | 173Gln | 0.880 | 0.044 | 0.859 | 0.043 | -14.23 | 1.00 |
| 303 | 174Gln | 0.886 | 0.044 | 0.807 | 0.040 | -13.45 | 0.67 |
| 304 | 175Met | 0.860 | 0.043 | 0.844 | 0.042 | -12.52 | 0.63 |
| 305 | 176Asn | 0.920 | 0.046 | 0.788 | 0.039 | -13.68 | 0.68 |
| 306[#] | 177Gly | 0.878 | 0.044 | 0.852 | 0.043 | -13.34 | 0.67 |
| 307[#] | 178Ser | 0.892 | 0.045 | 0.851 | 0.043 | -14.55 | 1.00 |

| | | | | | | | |
|---|---|---|---|---|---|---|---|
| 308[#] | 179Thr | 0.998 | 0.050 | 0.840 | 0.075 | -14.18 | 1.00 |
| 309[#] | 180Leu | 0.952 | 0.048 | 0.857 | 0.043 | -15.52 | 0.78 |
| 310[#] | 181Leu | 0.899 | 0.045 | 0.822 | 0.041 | -14.94 | 0.75 |
| 311[#] | 182Asn | 0.892 | 0.045 | 0.840 | 0.047 | -14.47 | 0.92 |
| 312 | 183Tyr | 0.946 | 0.047 | 0.866 | 0.043 | -14.88 | 0.74 |
| 313 | 184Ala | 0.985 | 0.049 | 0.866 | 0.044 | -13.88 | 0.79 |
| 314 | 185Arg | | | | | | |
| 315 | 186Phe | | | | | | |
| 316 | 187Arg | | | | | | |
| 317 | 188Asp | | | | | | |
| 318 | 189Asp | 0.907 | 0.045 | 0.829 | 0.053 | -14.28 | 0.71 |
| 319 | 190Asp | 0.919 | 0.046 | 0.829 | 0.041 | -12.13 | 0.89 |
| 320 | 191Glu | 0.924 | 0.046 | 0.858 | 0.043 | -13.74 | 1.00 |
| 321 | 192Ala | 0.987 | 0.049 | 0.857 | 0.043 | -13.39 | 1.00 |
| 322 | 193Asp | | | | | | |
| 323 | 194Tyr | | | | | | |
| 324[#] | 195Gly | 1.025 | 0.100 | 0.786 | 0.083 | -15.30 | 1.88 |
| 325[#] | 196Arg | 0.980 | 0.100 | 0.766 | 0.070 | -14.41 | 2.00 |
| 326 | 197Thr | | | | | | |
| 327 | 198Lys | | | | | | |
| 328[#] | 199Arg | 0.962 | 0.100 | 0.795 | 0.100 | -14.57 | 2.00 |
| 329[#] | 200Gln | 1.013 | 0.051 | 0.766 | 0.100 | -13.99 | 1.00 |
| 330 | 201Gln | | | | | | |
| 331[#] | 202Gln | 0.942 | 0.047 | 0.800 | 0.051 | -14.64 | 1.58 |
| 332 | 203Val | | | | | | |
| 333 | 204Leu | | | | | | |
| 334[#] | 205Thr | 1.000 | 0.050 | 0.851 | 0.070 | -14.39 | 1.00 |
| 335[#] | 206Ala | 0.940 | 0.047 | 0.864 | 0.043 | -14.60 | 0.73 |
| 336[#] | 207Ile | 0.862 | 0.043 | 0.874 | 0.044 | -15.47 | 0.87 |
| 337[#] | 208Leu | 0.920 | 0.046 | 0.771 | 0.054 | -14.51 | 0.73 |
| 338 | 209Glu | | | | | | |
| 339 | 210Gln | | | | | | |
| 340[#] | 211 Ile | 0.939 | 0.047 | 0.822 | 0.049 | -13.94 | 1.45 |
| 341[#] | 212Lys | 1.027 | 0.051 | 0.782 | 0.047 | -14.09 | 0.92 |
| 342 | 213Asp | | | | | | |
| 343 | 214Pro | | | | | | |
| 344 | 215Thr | 1.174 | 0.059 | 0.81 | 0.402 | -12.01 | 1.31 |
| 345 | 216Lys | | | | | | |
| 346 | 217Leu | | | | | | |
| 347 | 218Phe | | | | | | |
| 348 | 219Thr | | | | | | |
| 349 | 220Gly | | | | | | |
| 350 | 221 Ser | | | | | | |
| 351 | 222Glu | | | | | | |
| 352 | 223Ala | | | | | | |
| 353[#] | 224Leu | 0.915 | 0.103 | 0.728 | 0.145 | -12.52 | 3.00 |
| 354[#] | 225Gly | 0.951 | 0.103 | 0.802 | 0.079 | -13.19 | 1.88 |
| 355 | 226Lys | | | | | | |
| 356 | 227Val | | | | | | |
| 357 | 228Phe | | | | | | |
| 358 | 229Ala | | | | | | |
| 359 | 230Met | | | | | | |

| | | | | | | | |
|---|---|---|---|---|---|---|---|
| 360 | 231Thr | 0.954 | 0.064 | 0.731 | 0.150 | -12.87 | 3.00 |
| 361# | 232 Ser | 0.929 | 0.047 | 0.724 | 0.080 | -15.02 | 2.18 |
| 362# | 233Thr | 0.887 | 0.044 | 0.839 | 0.042 | -14.60 | 0.98 |
| 363 | 234Asn | | | | | | |
| 364# | 235Val | 0.915 | 0.046 | 0.824 | 0.041 | -14.91 | 0.75 |
| 365 | 236Pro | | | | | | |
| 366# | 237Tyr | 0.919 | 0.046 | 0.825 | 0.041 | -14.89 | 0.74 |
| 367# | 238Thr | 1.021 | 0.074 | 0.717 | 0.119 | -15.88 | 2.00 |
| 368 | 239Phe | | | | | | |
| 369 | 240Leu | | | | | | |
| 370 | 241Leu | | | | | | |
| 371 | 242Thr | | | | | | |
| 372 | 243Asn | | | | | | |
| 373# | 244Gly | 0.900 | 0.100 | 0.733 | 0.121 | -14.33 | 2.00 |
| 374# | 245Leu | 0.900 | 0.100 | 0.808 | 0.151 | -14.15 | 1.50 |
| 375# | 246 Ser | 0.987 | 0.049 | 0.840 | 0.104 | -13.39 | 1.64 |
| 376 | 247Val | | | | | | |
| 377 | 248Leu | | | | | | |
| 378 | 249Asp | | | | | | |
| 379 | 250Gly | | | | | | |
| 380# | 251Ala | 1.004 | 0.100 | 0.831 | 0.046 | -13.28 | 0.76 |
| 381# | 252Lys | 0.900 | 0.100 | 0.845 | 0.070 | -13.99 | 1.56 |
| 382 | 253Asn | 0.900 | 0.100 | 0.698 | 0.051 | -11.95 | 1.27 |
| 383 | 254Gly | 0.975 | 0.100 | 0.707 | 0.041 | -11.29 | 0.56 |
| 384 | 255 Ile | 1.029 | 0.100 | 0.712 | 0.044 | -11.25 | 1.60 |
| 385 | 256Glu | 0.909 | 0.045 | 0.800 | 0.040 | -13.71 | 0.69 |
| 386 | 257Lys | 0.905 | 0.045 | 0.782 | 0.039 | -12.86 | 0.64 |
| 387 | 258Leu | 0.925 | 0.046 | 0.722 | 0.036 | -12.96 | 0.65 |
| 388 | 259Thr | 0.983 | 0.049 | 0.769 | 0.038 | -14.70 | 0.74 |
| 389 | 260Ile | 0.922 | 0.046 | 0.847 | 0.042 | -12.84 | 0.64 |
| 390 | Pro | | | | | | |
| 391 | 262Glu | 0.910 | 0.045 | 0.818 | 0.041 | -12.73 | 0.64 |
| 392 | 263Leu | 0.858 | 0.043 | 0.726 | 0.053 | -13.67 | 0.68 |
| 393 | 264Gly | 0.874 | 0.044 | 0.665 | 0.063 | -11.07 | 0.55 |
| 394 | 265Asp | 0.977 | 0.049 | 0.728 | 0.036 | -14.59 | 0.73 |
| 395 | 266Trp | 0.856 | 0.043 | 0.834 | 0.042 | -12.92 | 0.65 |
| 396 | 267Val | 0.891 | 0.045 | 0.847 | 0.042 | -13.93 | 0.70 |
| 397 | 268Asp | 0.867 | 0.043 | 0.805 | 0.040 | -13.48 | 0.67 |
| 398 | 269Ala | 0.920 | 0.052 | 0.700 | 0.070 | -14.05 | 0.70 |
| 399 | 270Tyr | 0.900 | 0.100 | 0.667 | 0.033 | -10.57 | 0.53 |
| 400 | 271Asp | 1.050 | 0.100 | 0.693 | 0.080 | -10.73 | 2.18 |
| 401 | 272Val | 1.117 | 0.056 | 0.631 | 0.032 | -10.19 | 0.51 |
| 402 | 273Tyr | 1.061 | 0.053 | 0.700 | 0.035 | -11.28 | 1.00 |
| 403 | 274Gly | 1.050 | 0.053 | 0.692 | 0.070 | -13.10 | 1.00 |
| 404 | 275Gly | 1.000 | 0.050 | 0.626 | 0.060 | -13.54 | 1.00 |
| 405 | 276Leu | 0.933 | 0.047 | 0.685 | 0.034 | -12.51 | 0.63 |
| 406 | 277Gly | 0.838 | 0.042 | 0.729 | 0.036 | -13.14 | 0.66 |
| 407 | 278Leu | 0.908 | 0.045 | 0.782 | 0.046 | -15.08 | 0.75 |
| 408 | 279Leu | 0.880 | 0.044 | 0.757 | 0.047 | -15.81 | 0.79 |
| 409 | 280Val | 0.907 | 0.045 | 0.758 | 0.050 | -13.91 | 0.70 |
| 410 | 281Asp | 0.917 | 0.046 | 0.723 | 0.045 | -14.67 | 0.73 |
| 411 | 282Gln | 0.857 | 0.043 | 0.705 | 0.042 | -14.03 | 0.70 |

| | | | | | | | |
|---|---|---|---|---|---|---|---|
| 412 | 283Asn | 0.945 | 0.047 | 0.730 | 0.043 | -15.15 | 0.76 |
| 413 | 284Lys | 0.935 | 0.047 | 0.751 | 0.049 | -15.26 | 0.76 |
| 414 | 285Tyr | 0.935 | 0.047 | 0.759 | 0.038 | -14.87 | 0.74 |
| 415 | 286Gln | 0.905 | 0.045 | 0.816 | 0.041 | -15.36 | 0.77 |
| 416 | 287Thr | 0.900 | 0.045 | 0.811 | 0.060 | -15.14 | 1.50 |
| 417 | 288Lys | 0.912 | 0.050 | 0.854 | 0.043 | -15.69 | 0.78 |
| 418 | 289Leu | 0.903 | 0.045 | 0.865 | 0.043 | -15.30 | 0.77 |
| 419 | 290Ala | 0.904 | 0.045 | 0.864 | 0.043 | -15.69 | 0.78 |
| 420 | 291Gln | 0.922 | 0.046 | 0.844 | 0.042 | -14.91 | 0.75 |
| 421 | 292Met | 0.877 | 0.044 | 0.837 | 0.042 | -14.16 | 0.71 |
| 422 | 293Gly | 0.876 | 0.044 | 0.809 | 0.040 | -14.06 | 0.70 |
| 423 | 294Leu | 0.981 | 0.049 | 0.863 | 0.043 | -15.40 | 0.77 |
| 424 | 295Arg | 0.940 | 0.047 | 0.864 | 0.043 | -14.33 | 0.72 |

[#]amino acids with $S^2 > 0.8$, using for estimation of the $\tau_c$ value for the $Psr_{Sp}$ protein.

**Table S2** The population of the 10 best clusters obtained from trajectory segments (I) and (II) by cluster analyse of the $P_{srSp}$ protein

| **Number of cluster/ type of trajectory** | **1** | **2** | **3** | **4** | **5** | **6** | **7** | **8** | **9** | **10** | **others** |
|---|---|---|---|---|---|---|---|---|---|---|---|
| **Trajectory segment (I[a]) 700-1200ns** | 52.9% | 9.3% | 5.8% | 5.0% | 4.6% | 4.3% | 3.8% | 2.3% | 2.0% | 1.4% | 8.6% |
| **Trajectory segment (II[b]) 1750-2250ns** | 44.2% | 15.0% | 13.0% | 7.5% | 2.7% | 2.2% | 1.8% | 1.7% | 1.3% | 1.2% | 9.4% |

[a] For 700-1200 ns: the cluster analyses cut off was RMSD 0.095nm.
[b] For 1750-2250 ns: the cluster analyses cut off was RMSD 0.105nm.

**Table S3** Back calculated relaxation parameters $R_1$, $\eta_{xy}$ and NOE data with their errors ($\sigma$) obtained from trajectory **I** (700-1200ns) of the $P_{srSp}$ protein

| **N residue** | **Peak name** | **$R_1$ ($s^{-1}$)** | **$\sigma$ ($R_1$)** | **NOE** | **$\sigma$ (NOE)** | **$\eta_{xy}$ ($s^{-1}$)** | **$\sigma$ ($\eta_{xy}$)** |
|---|---|---|---|---|---|---|---|
| 130 | 1Gly | 0.91 | 0.059 | 0.288 | 0.042 | -8.246 | 0.82 |
| 131 | 2Glu | 0.887 | 0.06 | 0.474 | 0.057 | -9.199 | 0.953 |
| 132 | 3Val | 0.907 | 0.06 | 0.47 | 0.038 | -8.579 | 0.968 |
| 133 | 4Glu | 0.956 | 0.077 | 0.495 | 0.075 | -9.796 | 1.214 |
| 134 | 5Val | 0.918 | 0.11 | 0.661 | 0.039 | -9.445 | 1.022 |
| 135 | 6Phe | 1.004 | 0.091 | 0.675 | 0.03 | -11.614 | 1.183 |
| 136 | 7Asn | 0.892 | 0.038 | 0.678 | 0.015 | -12.06 | 0.712 |
| 137 | 8Gly | 0.795 | 0.043 | 0.346 | 0.074 | -12.273 | 1.5 |
| 138 | 9Gln | 0.883 | 0.033 | 0.767 | 0.009 | -13.228 | 0.763 |
| 139 | 10Asp | 0.853 | 0.032 | 0.783 | 0.007 | -13.278 | 0.759 |
| 140 | 11Thr | 0.818 | 0.031 | 0.767 | 0.013 | -12.622 | 0.731 |
| 141 | 12Arg | 0.871 | 0.032 | 0.744 | 0.01 | -13.27 | 0.758 |
| 142 | 13Asp | 0.919 | 0.036 | 0.74 | 0.01 | -12.357 | 0.732 |
| 143 | 14Gly | 0.864 | 0.032 | 0.759 | 0.021 | -13.034 | 0.777 |

| | | | | | | | |
|---|---|---|---|---|---|---|---|
| 144 | 15Val | 0.898 | 0.033 | 0.807 | 0.006 | -14.174 | 0.809 |
| 145 | 16Asn | 0.901 | 0.033 | 0.817 | 0.006 | -14.317 | 0.816 |
| 146 | 17Ile | 0.908 | 0.034 | 0.826 | 0.005 | -14.583 | 0.831 |
| 147 | 18Leu | 0.909 | 0.034 | 0.829 | 0.006 | -14.587 | 0.832 |
| 148 | 19Ile | 0.888 | 0.033 | 0.826 | 0.005 | -14.24 | 0.812 |
| 149 | 20Met | 0.902 | 0.033 | 0.818 | 0.005 | -14.324 | 0.817 |
| 150 | 21Gly | 0.88 | 0.033 | 0.791 | 0.015 | -13.782 | 0.807 |
| 151 | 22Thr | 0.904 | 0.033 | 0.786 | 0.018 | -14.039 | 0.804 |
| 152 | 23Asp | 0.917 | 0.034 | 0.75 | 0.014 | -13.565 | 0.777 |
| 153 | 24Gly | 1.051 | 0.068 | 0.678 | 0.017 | -11.817 | 0.769 |
| 154 | 25Arg | 0.955 | 0.044 | 0.547 | 0.023 | -9.212 | 0.629 |
| 155 | 26Ile | 1.122 | 0.078 | 0.415 | 0.029 | -7.154 | 0.737 |
| 156 | 27Gly | 1.199 | 0.082 | 0.424 | 0.039 | -6.486 | 0.469 |
| 157 | 28Gln | 1.16 | 0.058 | 0.394 | 0.046 | -5.549 | 0.423 |
| 158 | 29Asn | 1.208 | 0.068 | 0.369 | 0.035 | -5.235 | 0.363 |
| 159 | 30Ser | 1.28 | 0.054 | 0.339 | 0.029 | -4.089 | 0.263 |
| 160 | 31Val | 1.152 | 0.058 | 0.378 | 0.023 | -4.714 | 0.314 |
| 161 | 32Glu | 1.296 | 0.056 | 0.424 | 0.026 | -6.737 | 0.423 |
| 162 | 33Thr | 1.158 | 0.058 | 0.549 | 0.029 | -9.136 | 0.528 |
| 163 | 34Arg | 1.029 | 0.039 | 0.689 | 0.014 | -11.944 | 0.687 |
| 164 | 35Thr | 0.944 | 0.036 | 0.748 | 0.01 | -13.193 | 0.758 |
| 165 | 36Asp | 0.917 | 0.034 | 0.782 | 0.009 | -13.643 | 0.785 |
| 166 | 37Ser | 0.893 | 0.033 | 0.763 | 0.028 | -13.207 | 0.83 |
| 167 | 38Ile | 0.903 | 0.037 | 0.801 | 0.007 | -13.576 | 0.824 |
| 168 | 39Met | 0.914 | 0.034 | 0.826 | 0.005 | -14.6 | 0.832 |
| 169 | 40 Val | 0.902 | 0.033 | 0.809 | 0.006 | -14.367 | 0.819 |
| 170 | 41Leu | 0.909 | 0.034 | 0.831 | 0.006 | -14.61 | 0.833 |
| 171 | 42Asn | 0.893 | 0.033 | 0.823 | 0.005 | -14.303 | 0.815 |
| 172 | 43Val | 0.903 | 0.033 | 0.822 | 0.006 | -14.364 | 0.819 |
| 173 | 44Gly | 0.863 | 0.032 | 0.818 | 0.005 | -13.579 | 0.774 |
| 174 | 45Gly | 0.86 | 0.032 | 0.799 | 0.006 | -13.44 | 0.767 |
| 175 | 46Ser | 0.91 | 0.035 | 0.749 | 0.019 | -13.456 | 0.783 |
| 176 | 47Asp | 0.823 | 0.031 | 0.732 | 0.014 | -12.198 | 0.699 |
| 177 | 48Lys | 0.887 | 0.033 | 0.728 | 0.013 | -13.021 | 0.751 |
| 178 | 49Lys | 0.867 | 0.032 | 0.697 | 0.017 | -12.47 | 0.74 |
| 179 | 50Met | 0.887 | 0.033 | 0.776 | 0.008 | -13.542 | 0.773 |
| 180 | 51Lys | 0.877 | 0.033 | 0.792 | 0.008 | -13.594 | 0.775 |
| 181 | 52Leu | 0.907 | 0.034 | 0.821 | 0.005 | -14.444 | 0.823 |
| 182 | 53Val | 0.905 | 0.033 | 0.824 | 0.005 | -14.41 | 0.821 |
| 183 | 54Ser | 0.903 | 0.033 | 0.82 | 0.006 | -14.346 | 0.818 |
| 184 | 55Phe | 0.905 | 0.033 | 0.826 | 0.006 | -14.435 | 0.823 |
| 185 | 56Met | 0.908 | 0.034 | 0.806 | 0.007 | -14.038 | 0.801 |
| 186 | 57Arg | 0.902 | 0.033 | 0.807 | 0.006 | -13.977 | 0.797 |
| 187 | 58Asp | 0.912 | 0.034 | 0.823 | 0.005 | -14.425 | 0.822 |
| 188 | 59Asn | 0.905 | 0.033 | 0.819 | 0.005 | -14.309 | 0.816 |
| 189 | 60Leu | 0.903 | 0.033 | 0.817 | 0.006 | -14.228 | 0.811 |
| 190 | 61Val | 0.902 | 0.033 | 0.807 | 0.007 | -14.19 | 0.809 |
| 191 | 62Tyr | 0.908 | 0.034 | 0.798 | 0.007 | -13.935 | 0.796 |
| 192 | 63Ile | 0.911 | 0.034 | 0.782 | 0.01 | -13.988 | 0.801 |
| 193 | 64Asp | 0.899 | 0.033 | 0.778 | 0.009 | -13.762 | 0.785 |
| 194 | 65Gly | 0.809 | 0.03 | 0.751 | 0.01 | -12.192 | 0.695 |
| 195 | 66Tyr | 0.81 | 0.042 | 0.724 | 0.017 | -9.776 | 0.56 |

| | | | | | | | |
|---|---|---|---|---|---|---|---|
| 196 | 67Ser | 0.892 | 0.038 | 0.54 | 0.036 | -10.506 | 0.657 |
| 197 | 68Gln | 0.867 | 0.033 | 0.706 | 0.01 | -12.071 | 0.721 |
| 198 | 69Val | 0.918 | 0.035 | 0.64 | 0.018 | -11.122 | 0.741 |
| 199 | 70Ile | 0.994 | 0.067 | 0.636 | 0.021 | -11.832 | 0.808 |
| 200 | 71Asn | 0.903 | 0.034 | 0.611 | 0.029 | -11.805 | 0.687 |
| 201 | 72Gly | 0.893 | 0.034 | 0.624 | 0.026 | -11.483 | 0.665 |
| 202 | 73Arg | 0.889 | 0.04 | 0.595 | 0.015 | -10.933 | 0.652 |
| 203 | 74Lys | 0.916 | 0.034 | 0.602 | 0.015 | -11.885 | 0.68 |
| 204 | 75Gln | 0.894 | 0.033 | 0.619 | 0.018 | -11.746 | 0.704 |
| 205 | 76Thr | 0.909 | 0.046 | 0.563 | 0.021 | -10.768 | 0.663 |
| 206 | 77Asp | 0.887 | 0.033 | 0.606 | 0.074 | -12.082 | 0.852 |
| 207 | 78Asn | 0.905 | 0.034 | 0.755 | 0.019 | -12.987 | 0.801 |
| 208 | 79Lys | 0.895 | 0.033 | 0.808 | 0.006 | -13.819 | 0.788 |
| 209 | 80Leu | 0.913 | 0.034 | 0.821 | 0.006 | -14.371 | 0.819 |
| 210 | 81Asn | 0.915 | 0.034 | 0.821 | 0.006 | -14.485 | 0.826 |
| 211 | 82Val | 0.91 | 0.034 | 0.823 | 0.006 | -14.397 | 0.821 |
| 212 | 83Ala | 0.911 | 0.034 | 0.828 | 0.005 | -14.532 | 0.828 |
| 213 | 84Tyr | 0.917 | 0.034 | 0.826 | 0.006 | -14.491 | 0.826 |
| 214 | 85Glu | 0.927 | 0.034 | 0.821 | 0.006 | -14.469 | 0.825 |
| 215 | 86Leu | 0.929 | 0.034 | 0.822 | 0.006 | -14.621 | 0.833 |
| 216 | 87Gly | 0.911 | 0.034 | 0.821 | 0.006 | -14.34 | 0.817 |
| 217 | 88Glu | 0.922 | 0.034 | 0.81 | 0.007 | -14.374 | 0.82 |
| 218 | 89Gln | 0.921 | 0.035 | 0.713 | 0.012 | -13.576 | 0.776 |
| 219 | 90Glu | 0.896 | 0.034 | 0.725 | 0.012 | -13.234 | 0.758 |
| 220 | 91Gly | 0.856 | 0.035 | 0.531 | 0.032 | -10.012 | 0.655 |
| 221 | 92Gln | 0.994 | 0.041 | 0.518 | 0.026 | -10.889 | 0.65 |
| 222 | 93Lys | 0.94 | 0.036 | 0.656 | 0.014 | -12.116 | 0.697 |
| 223 | 94Gly | 0.91 | 0.034 | 0.771 | 0.007 | -13.962 | 0.796 |
| 224 | 95Ala | 0.895 | 0.033 | 0.799 | 0.006 | -14.023 | 0.799 |
| 225 | 96Glu | 0.912 | 0.034 | 0.822 | 0.006 | -14.5 | 0.827 |
| 226 | 97Met | 0.925 | 0.034 | 0.832 | 0.005 | -14.784 | 0.843 |
| 227 | 98Val | 0.919 | 0.034 | 0.829 | 0.005 | -14.701 | 0.838 |
| 228 | 99Arg | 0.925 | 0.034 | 0.832 | 0.005 | -14.809 | 0.844 |
| 229 | 100Gln | 0.923 | 0.034 | 0.83 | 0.005 | -14.816 | 0.845 |
| 230 | 101Val | 0.922 | 0.034 | 0.832 | 0.005 | -14.784 | 0.843 |
| 231 | 102Leu | 0.926 | 0.034 | 0.835 | 0.005 | -14.817 | 0.845 |
| 232 | 103Lys | 0.922 | 0.034 | 0.832 | 0.005 | -14.705 | 0.838 |
| 233 | 104Asp | 0.919 | 0.034 | 0.831 | 0.005 | -14.646 | 0.835 |
| 234 | 105Asn | 0.892 | 0.033 | 0.807 | 0.007 | -13.906 | 0.793 |
| 235 | 106Phe | 0.88 | 0.033 | 0.81 | 0.006 | -13.785 | 0.786 |
| 236 | 107Asp | 0.892 | 0.033 | 0.813 | 0.006 | -14.058 | 0.802 |
| 237 | 108Leu | 0.825 | 0.031 | 0.721 | 0.013 | -12.256 | 0.7 |
| 238 | 109Asp | 0.89 | 0.033 | 0.808 | 0.008 | -14.104 | 0.805 |
| 239 | 110Ile | 0.882 | 0.033 | 0.803 | 0.009 | -13.72 | 0.789 |
| 240 | 111Lys | 0.894 | 0.033 | 0.817 | 0.006 | -14.06 | 0.802 |
| 241 | 112Tyr | 0.882 | 0.033 | 0.824 | 0.006 | -13.967 | 0.799 |
| 242 | 113Tyr | 0.912 | 0.034 | 0.829 | 0.005 | -14.615 | 0.833 |
| 243 | 114Ala | 0.909 | 0.034 | 0.83 | 0.006 | -14.559 | 0.83 |
| 244 | 115Leu | 0.895 | 0.033 | 0.826 | 0.006 | -14.276 | 0.814 |
| 245 | 116Val | 0.908 | 0.034 | 0.819 | 0.008 | -14.312 | 0.817 |
| 246 | 117Asp | 0.894 | 0.033 | 0.804 | 0.007 | -13.886 | 0.793 |
| 247 | 118Phe | 0.902 | 0.033 | 0.816 | 0.006 | -14.339 | 0.817 |

| | | | | | | | |
|---|---|---|---|---|---|---|---|
| 248 | 119Gln | 0.894 | 0.033 | 0.79 | 0.007 | -14.01 | 0.799 |
| 249 | 120Ala | 0.861 | 0.032 | 0.798 | 0.007 | -13.544 | 0.772 |
| 250 | 121Phe | 0.866 | 0.032 | 0.799 | 0.009 | -13.529 | 0.774 |
| 251 | 122Ala | 0.916 | 0.034 | 0.829 | 0.006 | -14.684 | 0.837 |
| 252 | 123Thr | 0.907 | 0.033 | 0.819 | 0.006 | -14.487 | 0.826 |
| 253 | 124Ala | 0.909 | 0.034 | 0.825 | 0.006 | -14.565 | 0.83 |
| 254 | 125 Ile | 0.921 | 0.034 | 0.828 | 0.005 | -14.739 | 0.84 |
| 255 | 126Asp | 0.917 | 0.034 | 0.828 | 0.005 | -14.661 | 0.836 |
| 256 | 127Thr | 0.874 | 0.032 | 0.819 | 0.006 | -13.982 | 0.798 |
| 257 | 128Leu | 0.908 | 0.034 | 0.821 | 0.005 | -14.418 | 0.822 |
| 258 | 129Phe | 0.907 | 0.034 | 0.821 | 0.006 | -14.354 | 0.818 |
| 259 | 130Pro | | | | | | |
| 260 | 131Asp | 0.859 | 0.032 | 0.799 | 0.008 | -13.142 | 0.75 |
| 261 | 132Gly | 0.839 | 0.031 | 0.808 | 0.006 | -13.058 | 0.745 |
| 262 | 133Val | 0.911 | 0.034 | 0.789 | 0.008 | -13.473 | 0.774 |
| 263 | 134Thr | 0.923 | 0.035 | 0.81 | 0.007 | -13.617 | 0.778 |
| 264 | 135Ile | 0.914 | 0.034 | 0.817 | 0.007 | -14.103 | 0.806 |
| 265 | 136Asp | 0.894 | 0.033 | 0.787 | 0.008 | -13.392 | 0.769 |
| 266 | 137Ala | 0.927 | 0.038 | 0.757 | 0.014 | -12.686 | 0.724 |
| 267 | 138Gln | 0.922 | 0.034 | 0.757 | 0.011 | -13.3 | 0.762 |
| 268 | 139Phe | 0.909 | 0.034 | 0.722 | 0.012 | -12.582 | 0.72 |
| 269 | 140Ser | 0.949 | 0.036 | 0.766 | 0.011 | -13.484 | 0.77 |
| 270 | 141Thr | 0.931 | 0.065 | 0.698 | 0.022 | -10.28 | 1.207 |
| 271 | 142Leu | 0.991 | 0.059 | 0.777 | 0.008 | -13.521 | 0.848 |
| 272 | 143Asn | 0.933 | 0.037 | 0.678 | 0.014 | -12.826 | 0.732 |
| 273 | 144Gly | 0.936 | 0.038 | 0.727 | 0.012 | -13.211 | 0.757 |
| 274 | 145Arg | 0.853 | 0.038 | 0.764 | 0.008 | -12.005 | 0.692 |
| 275 | 146Pro | | | | | | |
| 276 | 147Leu | 0.936 | 0.046 | 0.717 | 0.017 | -10.091 | 0.731 |
| 277 | 148Thr | 0.927 | 0.039 | 0.771 | 0.011 | -12.495 | 0.723 |
| 278 | 149Glu | 0.878 | 0.033 | 0.754 | 0.008 | -13.07 | 0.747 |
| 279 | 150Ala | 0.904 | 0.034 | 0.781 | 0.009 | -13.538 | 0.778 |
| 280 | 151Thr | 0.862 | 0.035 | 0.745 | 0.016 | -12.403 | 0.726 |
| 281 | 152Val | 0.962 | 0.054 | 0.722 | 0.024 | -12.673 | 0.845 |
| 282 | 153Gly | 1.056 | 0.046 | 0.61 | 0.086 | -9.727 | 0.632 |
| 283 | 154Asp | 1.121 | 0.053 | 0.437 | 0.059 | -7.88 | 0.584 |
| 284 | 155Asp | 1.306 | 0.059 | 0.242 | 0.039 | -5.148 | 0.402 |
| 285 | 156Leu | 1.365 | 0.058 | 0.244 | 0.055 | -5.348 | 0.422 |
| 286 | 157Tyr | 1.366 | 0.056 | 0.221 | 0.032 | -4.609 | 0.372 |
| 287 | 158Ala | 1.209 | 0.054 | 0.139 | 0.06 | -4.696 | 0.383 |
| 288 | 159Thr | 1.18 | 0.049 | 0.148 | 0.071 | -3.584 | 0.264 |
| 289 | 160Glu | 1.284 | 0.059 | 0.124 | 0.057 | -3.913 | 0.27 |
| 290 | 161Thr | 1.097 | 0.049 | 0.088 | 0.054 | -4.377 | 0.346 |
| 291 | 162Glu | 1.14 | 0.064 | 0.055 | 0.034 | -4.51 | 0.433 |
| 292 | 163Ser | 1.081 | 0.06 | 0.14 | 0.037 | -5.557 | 0.548 |
| 293 | 164Pro | | | | | | |
| 294 | 165Thr | 1.141 | 0.085 | 0.332 | 0.085 | -7.431 | 0.924 |
| 295 | 166Gln | 1.275 | 0.143 | 0.52 | 0.047 | -8.795 | 1.512 |
| 296 | 167Thr | 1.129 | 0.092 | 0.672 | 0.019 | -11.115 | 0.996 |
| 297 | 168Ile | 0.925 | 0.038 | 0.735 | 0.013 | -13.112 | 0.757 |
| 298 | 169Lys | 0.893 | 0.038 | 0.707 | 0.01 | -12.558 | 0.718 |
| 299 | 170Val | 0.924 | 0.038 | 0.76 | 0.016 | -13.202 | 0.789 |

| | | | | | | | |
|---|---|---|---|---|---|---|---|
| 300 | 171Gly | 0.909 | 0.035 | 0.75 | 0.011 | -12.806 | 0.735 |
| 301 | 172Lys | 0.879 | 0.033 | 0.74 | 0.013 | -12.747 | 0.729 |
| 302 | 173Gln | 0.907 | 0.034 | 0.749 | 0.019 | -13.306 | 0.798 |
| 303 | 174Gln | 0.888 | 0.033 | 0.767 | 0.014 | -13.025 | 0.787 |
| 304 | 175Met | 0.887 | 0.033 | 0.78 | 0.007 | -12.983 | 0.74 |
| 305 | 176Asn | 0.888 | 0.033 | 0.818 | 0.006 | -14.054 | 0.802 |
| 306 | 177Gly | 0.914 | 0.034 | 0.825 | 0.005 | -14.628 | 0.834 |
| 307 | 178Ser | 0.91 | 0.034 | 0.826 | 0.006 | -14.565 | 0.83 |
| 308 | 179Thr | 0.903 | 0.033 | 0.821 | 0.006 | -14.438 | 0.823 |
| 309 | 180Leu | 0.926 | 0.034 | 0.832 | 0.005 | -14.876 | 0.848 |
| 310 | 181Leu | 0.921 | 0.034 | 0.83 | 0.005 | -14.772 | 0.842 |
| 311 | 182Asn | 0.921 | 0.034 | 0.825 | 0.005 | -14.755 | 0.841 |
| 312 | 183Tyr | 0.923 | 0.034 | 0.833 | 0.005 | -14.83 | 0.845 |
| 313 | 184Ala | 0.92 | 0.034 | 0.834 | 0.005 | -14.761 | 0.841 |
| 314 | 185Arg | 0.903 | 0.033 | 0.828 | 0.005 | -14.436 | 0.823 |
| 315 | 186Phe | 0.877 | 0.032 | 0.808 | 0.008 | -13.702 | 0.786 |
| 316 | 187Arg | 0.909 | 0.034 | 0.805 | 0.006 | -14.194 | 0.809 |
| 317 | 188Asp | 0.886 | 0.033 | 0.806 | 0.007 | -13.717 | 0.783 |
| 318 | 189Asp | 0.851 | 0.033 | 0.172 | 0.098 | -9.237 | 0.588 |
| 319 | 190Asp | 0.935 | 0.035 | 0.761 | 0.011 | -14.032 | 0.8 |
| 320 | 191Glu | 0.915 | 0.037 | 0.729 | 0.011 | -12.85 | 0.734 |
| 321 | 192Ala | 0.91 | 0.034 | 0.796 | 0.007 | -14.053 | 0.802 |
| 322 | 193Asp | 0.894 | 0.033 | 0.821 | 0.005 | -14.051 | 0.801 |
| 323 | 194Tyr | 0.915 | 0.034 | 0.828 | 0.005 | -14.536 | 0.829 |
| 324 | 195Gly | 0.916 | 0.034 | 0.826 | 0.005 | -14.552 | 0.829 |
| 325 | 196Arg | 0.924 | 0.034 | 0.83 | 0.005 | -14.686 | 0.837 |
| 326 | 197Thr | 0.919 | 0.034 | 0.829 | 0.005 | -14.676 | 0.837 |
| 327 | 198Lys | 0.922 | 0.034 | 0.83 | 0.005 | -14.748 | 0.841 |
| 328 | 199Arg | 0.916 | 0.034 | 0.831 | 0.005 | -14.659 | 0.836 |
| 329 | 200Gln | 0.917 | 0.034 | 0.818 | 0.008 | -14.516 | 0.828 |
| 330 | 201Gln | 0.915 | 0.034 | 0.832 | 0.005 | -14.668 | 0.836 |
| 331 | 202Gln | 0.912 | 0.034 | 0.828 | 0.005 | -14.589 | 0.832 |
| 332 | 203Val | 0.923 | 0.034 | 0.83 | 0.005 | -14.771 | 0.842 |
| 333 | 204Leu | 0.924 | 0.034 | 0.834 | 0.005 | -14.779 | 0.842 |
| 334 | 205Thr | 0.916 | 0.034 | 0.828 | 0.006 | -14.668 | 0.836 |
| 335 | 206Ala | 0.92 | 0.034 | 0.828 | 0.005 | -14.724 | 0.839 |
| 336 | 207Ile | 0.915 | 0.034 | 0.831 | 0.005 | -14.642 | 0.835 |
| 337 | 208Leu | 0.913 | 0.034 | 0.828 | 0.005 | -14.586 | 0.832 |
| 338 | 209Glu | 0.909 | 0.034 | 0.819 | 0.006 | -14.393 | 0.82 |
| 339 | 210Gln | 0.906 | 0.034 | 0.811 | 0.008 | -14.011 | 0.799 |
| 340 | 211 Ile | 0.912 | 0.034 | 0.788 | 0.009 | -13.439 | 0.766 |
| 341 | 212Lys | 0.895 | 0.033 | 0.713 | 0.018 | -12.331 | 0.722 |
| 342 | 213Asp | 0.899 | 0.04 | 0.734 | 0.012 | -12.053 | 0.74 |
| 343 | 214Pro | | | | | | |
| 344 | 215Thr | 0.914 | 0.034 | 0.79 | 0.007 | -13.797 | 0.787 |
| 345 | 216Lys | 0.909 | 0.034 | 0.762 | 0.009 | -13.198 | 0.758 |
| 346 | 217Leu | 0.924 | 0.035 | 0.783 | 0.011 | -13.521 | 0.773 |
| 347 | 218Phe | 0.947 | 0.035 | 0.797 | 0.008 | -13.908 | 0.794 |
| 348 | 219Thr | 0.944 | 0.036 | 0.747 | 0.014 | -12.57 | 0.737 |
| 349 | 220Gly | 1.198 | 0.066 | 0.537 | 0.045 | -8.43 | 0.935 |
| 350 | 221 Ser | 1.134 | 0.092 | 0.386 | 0.08 | -7.122 | 0.439 |
| 351 | 222Glu | 0.945 | 0.037 | 0.699 | 0.016 | -12.598 | 0.726 |

| | | | | | | | |
|---|---|---|---|---|---|---|---|
| 352 | 223Ala | 0.955 | 0.039 | 0.71 | 0.021 | -12.9 | 0.78 |
| 353 | 224Leu | 0.925 | 0.034 | 0.795 | 0.007 | -14.054 | 0.805 |
| 354 | 225Gly | 0.913 | 0.034 | 0.805 | 0.009 | -14.064 | 0.803 |
| 355 | 226Lys | 0.909 | 0.034 | 0.819 | 0.006 | -14.151 | 0.809 |
| 356 | 227Val | 0.924 | 0.035 | 0.81 | 0.007 | -14.184 | 0.809 |
| 357 | 228Phe | 0.928 | 0.035 | 0.814 | 0.007 | -14.354 | 0.819 |
| 358 | 229Ala | 0.893 | 0.034 | 0.801 | 0.008 | -13.71 | 0.783 |
| 359 | 230Met | 0.902 | 0.036 | 0.741 | 0.012 | -13.223 | 0.755 |
| 360 | 231Thr | 0.872 | 0.033 | 0.782 | 0.009 | -13.337 | 0.762 |
| 361 | 232 Ser | 0.873 | 0.032 | 0.787 | 0.009 | -13.425 | 0.766 |
| 362 | 233Thr | 0.885 | 0.033 | 0.808 | 0.011 | -13.981 | 0.8 |
| 363 | 234Asn | 0.892 | 0.033 | 0.823 | 0.007 | -14.119 | 0.806 |
| 364 | 235Val | 0.903 | 0.033 | 0.828 | 0.005 | -14.301 | 0.816 |
| 365 | 236Pro | | | | | | |
| 366 | 237Tyr | 0.928 | 0.035 | 0.809 | 0.01 | -13.708 | 0.794 |
| 367 | 238Thr | 0.954 | 0.036 | 0.815 | 0.007 | -13.675 | 0.79 |
| 368 | 239Phe | 0.909 | 0.034 | 0.817 | 0.006 | -14.206 | 0.81 |
| 369 | 240Leu | 0.934 | 0.035 | 0.815 | 0.006 | -14.131 | 0.806 |
| 370 | 241Leu | 0.932 | 0.035 | 0.822 | 0.005 | -14.227 | 0.812 |
| 371 | 242Thr | 0.919 | 0.034 | 0.782 | 0.009 | -13.558 | 0.784 |
| 372 | 243Asn | 0.938 | 0.038 | 0.786 | 0.012 | -12.865 | 0.766 |
| 373 | 244Gly | 0.882 | 0.033 | 0.809 | 0.006 | -13.517 | 0.771 |
| 374 | 245Leu | 0.913 | 0.034 | 0.806 | 0.006 | -14.034 | 0.801 |
| 375 | 246 Ser | 0.923 | 0.035 | 0.813 | 0.006 | -13.998 | 0.801 |
| 376 | 247Val | 0.92 | 0.034 | 0.821 | 0.005 | -14.501 | 0.827 |
| 377 | 248Leu | 0.92 | 0.034 | 0.814 | 0.006 | -14.46 | 0.824 |
| 378 | 249Asp | 0.925 | 0.034 | 0.814 | 0.006 | -14.483 | 0.826 |
| 379 | 250Gly | 0.91 | 0.034 | 0.819 | 0.007 | -14.414 | 0.822 |
| 380 | 251Ala | 0.916 | 0.034 | 0.818 | 0.007 | -14.51 | 0.827 |
| 381 | 252Lys | 0.914 | 0.034 | 0.816 | 0.006 | -14.437 | 0.823 |
| 382 | 253Asn | 0.886 | 0.033 | 0.817 | 0.006 | -13.947 | 0.796 |
| 383 | 254Gly | 0.88 | 0.033 | 0.769 | 0.01 | -13.033 | 0.751 |
| 384 | 255 Ile | 0.896 | 0.034 | 0.776 | 0.011 | -13.183 | 0.762 |
| 385 | 256Glu | 0.905 | 0.033 | 0.791 | 0.007 | -14.166 | 0.808 |
| 386 | 257Lys | 0.863 | 0.032 | 0.806 | 0.006 | -13.612 | 0.776 |
| 387 | 258Leu | 0.858 | 0.032 | 0.801 | 0.006 | -13.419 | 0.765 |
| 388 | 259Thr | 0.875 | 0.032 | 0.813 | 0.006 | -13.772 | 0.786 |
| 389 | 260Ile | 0.912 | 0.034 | 0.823 | 0.005 | -14.473 | 0.825 |
| 390 | Pro | | | | | | |
| 391 | 262Glu | 0.945 | 0.035 | 0.752 | 0.01 | -13.081 | 0.747 |
| 392 | 263Leu | 0.948 | 0.035 | 0.708 | 0.011 | -12.774 | 0.729 |
| 393 | 264Gly | 0.877 | 0.033 | 0.674 | 0.015 | -12.228 | 0.709 |
| 394 | 265Asp | 0.909 | 0.034 | 0.774 | 0.008 | -13.64 | 0.778 |
| 395 | 266Trp | 0.925 | 0.036 | 0.7 | 0.025 | -11.917 | 0.686 |
| 396 | 267Val | 0.941 | 0.035 | 0.733 | 0.01 | -13.264 | 0.757 |
| 397 | 268Asp | 0.964 | 0.043 | 0.655 | 0.016 | -11.193 | 0.649 |
| 398 | 269Ala | 0.959 | 0.068 | 0.498 | 0.033 | -7.755 | 0.573 |
| 399 | 270Tyr | 0.928 | 0.064 | 0.461 | 0.077 | -7.638 | 0.511 |
| 400 | 271Asp | 1.047 | 0.091 | 0.419 | 0.034 | -8.261 | 0.555 |
| 401 | 272Val | 1.148 | 0.094 | 0.318 | 0.05 | -7.126 | 0.505 |
| 402 | 273Tyr | 1.147 | 0.086 | 0.349 | 0.065 | -7.28 | 0.611 |
| 403 | 274Gly | 1.136 | 0.103 | 0.295 | 0.054 | -5.87 | 0.791 |

| | | | | | | | |
|---|---|---|---|---|---|---|---|
| 404 | 275Gly | 1.129 | 0.101 | 0.408 | 0.051 | -5.886 | 0.502 |
| 405 | 276Leu | 1.055 | 0.069 | 0.475 | 0.021 | -8.772 | 0.597 |
| 406 | 277Gly | 1.1 | 0.108 | 0.532 | 0.028 | -8.38 | 0.76 |
| 407 | 278Leu | 0.897 | 0.034 | 0.753 | 0.008 | -13.02 | 0.744 |
| 408 | 279Leu | 0.895 | 0.033 | 0.771 | 0.008 | -13.452 | 0.769 |
| 409 | 280Val | 0.923 | 0.035 | 0.747 | 0.01 | -13.164 | 0.76 |
| 410 | 281Asp | 0.947 | 0.035 | 0.701 | 0.011 | -12.77 | 0.73 |
| 411 | 282Gln | 0.931 | 0.035 | 0.669 | 0.01 | -12.306 | 0.702 |
| 412 | 283Asn | 0.951 | 0.039 | 0.665 | 0.015 | -11.839 | 0.682 |
| 413 | 284Lys | 0.942 | 0.036 | 0.698 | 0.017 | -12.343 | 0.705 |
| 414 | 285Tyr | 0.92 | 0.034 | 0.776 | 0.01 | -13.874 | 0.792 |
| 415 | 286Gln | 0.907 | 0.033 | 0.818 | 0.006 | -14.198 | 0.809 |
| 416 | 287Thr | 0.917 | 0.034 | 0.813 | 0.006 | -14.267 | 0.813 |
| 417 | 288Lys | 0.913 | 0.034 | 0.796 | 0.006 | -14.076 | 0.802 |
| 418 | 289Leu | 0.927 | 0.034 | 0.818 | 0.006 | -14.533 | 0.829 |
| 419 | 290Ala | 0.924 | 0.034 | 0.816 | 0.006 | -14.47 | 0.825 |
| 420 | 291Gln | 0.919 | 0.034 | 0.809 | 0.006 | -14.343 | 0.818 |
| 421 | 292Met | 0.903 | 0.033 | 0.803 | 0.006 | -14.032 | 0.8 |
| 422 | 293Gly | 0.881 | 0.033 | 0.734 | 0.01 | -12.714 | 0.725 |
| 423 | 294Leu | 0.911 | 0.035 | 0.726 | 0.012 | -12.862 | 0.741 |
| 424 | 295Arg | 0.896 | 0.036 | 0.688 | 0.012 | -11.652 | 0.717 |

## Supplementary File S2

### Example of $^{1}H$-$^{15}N$ CSA/DD cross correlation ($\eta_{xy}$).

In **Figure S7(a)**, the first 2D plane at delay $\zeta$ equal to 0 in the $\eta_{xy}$ experiment for the $P_{srSp}$ protein is presented, with the experiment performed at a constant time interval of T = 60 ms. A comparison of the first increment of the $\eta_{xy}$ experiment (**Figure S7(e)**) with (black curve) and without (red dashed curve) water flip-back selective pulses show that implementing these pulses in the pulse sequence (**Figure 6**) prevents saturation of amino proton signals by water and improves the intensities of the observed signals by more than 1.5 times.

At constant time delay T=60, the intensity of the signal, shown in the projection through the 306G cross-peak, decreases approximately 2.5-fold, as evidenced by the comparison of the black curve (first 2D plane) and the red dashed curve (last 2D plane) in **Figure S7 (f)** for $\zeta$ delays of 0 and 0.098 s, respectively.

As an example, to illustrate the quality of the experimental data obtained under the aforementioned conditions, the intensity changes as a function of delay $\zeta$, fitted to exponential

curves to determine the $\eta_{xy}$ relaxation rates for the selected residues 174G, 307S, and 306G, are shown in **Figures S7(b)**, **(c)**, and **(d)**, respectively.

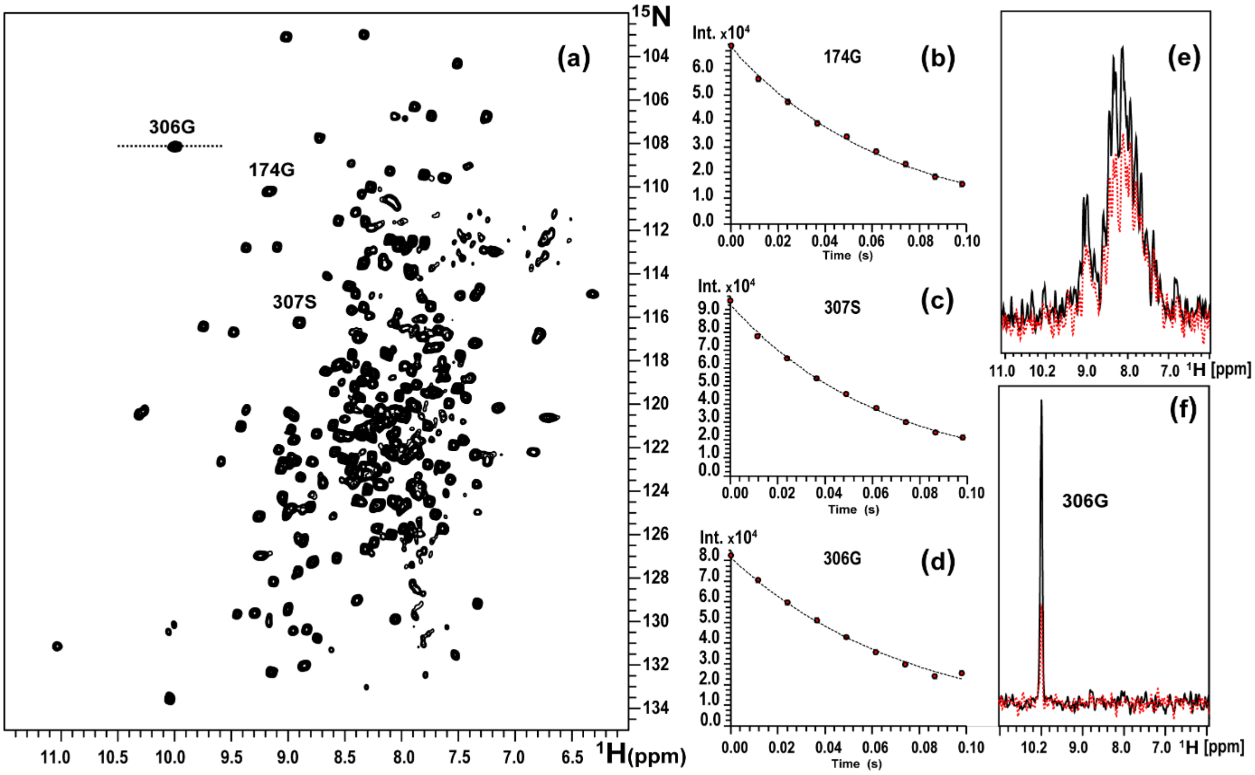


**Figure S7. Spectrum of $^{1}$H-$^{15}$N chemical shift anisotropy/dipole-dipole (CSA/DD) cross-correlations, $\eta_{xy}$, obtained on $P_{srSp}$ protein.**

(a) First 2D plane at ζ delay equal to 0 of the $\eta_{xy}$ experiment for the $P_{srSp}$ protein; (b), (c), and (d) visualization of intensity changes as a function of ζ delay, fitted with exponential curves to determine the $\eta_{xy}$ relaxation rates for the selected residues 174G, 307S, and 306G, respectively, as indicated in (a); (e) first increment of the $\eta_{xy}$ experiment with (black curve) and without (red dashed curve) water flip-back selective pulses, used to prevent saturation of amino proton signals by water; (f) projection through the 306G cross-peak (labelled in (a)), shown as a black curve (first 2D plane) and a red dashed curve (last 2D plane) for ζ delays of 0 and 0.098 s, respectively.
